# Variability in Morphological Traits and Nutritional Profiles of Adult *Eriocheir sinensis* in Different Aquacultural Regions

**DOI:** 10.3390/ani15020243

**Published:** 2025-01-16

**Authors:** Wenrong Feng, Qinghong He, Jianlin Li, Jun Zhou, Guoan Hua, Yuanfeng Xu, Gang Jiang, Yongkai Tang

**Affiliations:** 1 Key Laboratory of Freshwater Fisheries and Germplasm Resources Utilization, Ministry of Agriculture and Rural Affairs, Freshwater Fisheries Research Center, Chinese Academy of Fishery Sciences, Wuxi 214081, China; fengwenrong@ffrc.cn (W.F.); hqh13990750864@163.com (Q.H.); lijl@ffrc.cn (J.L.); xuyuanfeng@ffrc.cn (Y.X.); jianggang@ffrc.cn (G.J.); 2 Freshwater Fisheries Research Institute of Jiangsu Province, Nanjing 210017, China; finedrizzle@163.com; 3 China Jiangsu Haorun Biological Industry Group Co., Ltd., Taizhou 225311, China; 13338888115@163.com; 4 Wuxi Fisheries College, Nanjing Agricultural University, Wuxi 214081, China

**Keywords:** *Eriocheir sinensis*, aquaculture, morphometrics, amino acid, fatty acid

## Abstract

*Eriocheir sinensis* is a species of significant economic importance in China’s aquaculture industry. In order to investigate the variations in morphologic and nutritional profiles of farmed crabs across diverse farming regions, *E. sinensis* were collected from six aquafarms located in the primary cultivation areas in China. The findings revealed significant variations in specific morphological indices, but these are insufficient for clear regional distinction using PCA. Essential amino acid (EAA) levels varied by tissue and gender, with ovaries from the YY (Yongyan in Anhui province) group showing the highest levels, while the hepatopancreas in females from the JX (Jianxian in Jiangxi province) group had the lowest. The analysis of fatty acid contents revealed that muscles were rich in PUFAs, whereas MUFAs were dominant in the hepatopancreas and gonads. The fatty acid levels were significantly higher in ovaries than in testes, notably in the HZ (Huzhou in Zhejiang province), EZ (Ezhou in Hubei province), YY, and JX groups. Furthermore, the PCA based on proximate composition, amino acids, and fatty acids revealed distinct categorizations of *E. sinensis* from various regions. In conclusion, these findings highlight significant regional variations in the morphology and nutritional composition of farmed *E. sinensis*, suggesting the need for tailored aquaculture practices to optimize quality and output.

## Simple Summary

This study examines the differences in the body shape and nutrition of the Chinese mitten crab (*Eriocheir sinensis*) across various farming regions in China. Samples from six aquafarms showed differences in several morphological indices; however, these traits were not effective in distinguishing crabs from different regions. Nutritionally, this study found diverse levels of crude proteins, crude lipids, ash, and sugar in the muscle, hepatopancreas, and gonads. Notably, proteins were most abundant in reproductive tissues, while the hepatopancreas had the highest fat content. These crabs were rich in essential amino acids and unsaturated fatty acids, making them a good nutritional source. Principal component analysis (PCA) identified distinct categorizations of proximate composition, amino acids, and fatty acids in *E. sinensis* from various regions, which may be attributable to varied aquaculture conditions. This research enriches our understanding of regional variance in nutritional compositions of *E. sinensis* and enhances consumer awareness about the species.

## 1. Introduction

*Eriocheir sinensis* is a key species in China’s aquaculture due to its economic significance, which stems from its widespread popularity as a food item celebrated for its delicious meat and high nutritional value. Successful advancements in artificial propagation technology for *E. sinensis* spurred significant progress in breeding practices throughout the 1990s [1]. Since 2002, the industry has transitioned into a phase of ecological and efficient crab farming [2,3]. Over the past two decades, a mature breeding technology system for *E. sinensis* has been established and continuously refined, with both its scale and popularity consistently expanding [4]. The annual production of *E. sinensis* showed a generally increasing trend from 2003 to 2023, reaching a production volume of 888, 629 tons in 2023 [5]. The major provinces involved in the cultivation of *E. sinensis* include Jiangsu, Hubei, Anhui, Liaoning, Shandong, Jiangxi, Heilongjiang, and Zhejiang. However, significant variations in farming conditions exist for *E. sinensis* from different regions. As a result, exploring regional variations in the nutritional composition of *E. sinensis* has garnered more attention.

Crustaceans are an excellent source of high-quality protein and polyunsaturated fatty acids, making them a valuable dietary choice. *E. sinensis*, in particular, is rich in protein, omega-3 fatty acids, and essential minerals such as calcium, phosphorus, and iron. It is worth noting that the hepatopancreas of the crab, along with its paste (testes) and roe (eggs), are also considered delicacies [6]. The quality of crabs is one of the key factors that influences their market price. It has been reported that nutritional analysis is a commonly employed method for assessing the quality of *E. sinensis* [7]. In nutritional composition, amino acid and fatty acid contents directly reflect the nutritional quality of *E. sinensis* and are closely associated with its flavor and texture. These parameters have been employed to assess the nutritional quality of *E. sinensis* in the Yangtze River Delta region [8]. The nutritional composition of *E. sinensis* can vary depending on several factors, including habitat [9], size [10], the status of gonadal development [11], diet composition [12,13], carapace color [14], etc. For example, Zhang et al. reported variations in the amino acid and fatty acid contents of *E. sinensis* at different growth stages in both female and male sexes [15,16]. Lots of studies have focused on nutritional differences between wild-caught and farmed crabs. Wu et al. indicated that wild-caught crabs showed superior qualities in terms of minerals, fatty acids, and flavor compared to rice-field crabs [17]. An analysis of the edible portions of *E. sinensis* from wild-caught crabs and two groups of pond-reared crabs revealed that wild-caught specimens possessed the highest umami intensity, total free amino acid content, and key aromatic compounds [18]. A comparison of wild adult male *E. sinensis* muscles from five different rivers demonstrated significant variations in meat yield and flavor profiles among the geographic populations [19]. These variations might be attributed to genetic factors and the environmental conditions where each species develops [17,20,21]. Additionally, Wang et al. compared the nutritional variations of *E. sinensis* from four northern geographic regions under different farming models (paddies, lakes, and ponds) [22]. In the study of Mei et al., *E. sinensis* from rice fields were compared with those cultured in ponds based on several indexes, including fatty acids, and demonstrated that both groups exhibited good nutritional values, though rice-field crabs exhibited better qualities [23]. Meanwhile, Shang et al. examined the quality of *E. sinensis* from Yangtze River and Yellow River populations cultured in ponds by measuring their proximate composition, fatty acid, and amino acid profiles and found that there were some differences in the fatty acid and amino acid compositions [24]. Despite the many literatures available, there is still a lack of systematic research concerning regional variations in the quality of pond-reared *E. sinensis* across major aquaculture regions in China.

Across various regions, the biochemical composition of farmed *E. sinensis* may differ, and furthermore, due to variances in growth environments and diet compositions, their morphological characteristics may also vary. Morphological traits are an important parameter for species identification and an important improvement index in breeding work [25,26]. Currently, studies on the morphological traits of wild river crabs are extensive [25,27]. He et al. discussed the morphological differences between the populations of *E. sinensis* in the Liaohe River and Oujiang River [28]. Lu et al. showed that there were significant differences in the morphology and biochemical composition of wild *E. sinensis* populations from the Yangtze River, Liaohe River, and Yellow River [29]. Studies also indicated that when wild crabs from different river systems were cultivated under uniform conditions, these differences tended to diminish [30,31]. However, research is still limited on the morphological characteristics among farmed *E. sinensis* in diverse environmental conditions.

This study focuses on adult pond-reared *E. sinensis* across various regions in China. Our objective is to analyze variations in the morphometric features and biochemical composition of three edible parts (muscle, hepatopancreas, and gonad) of *E. sinensis* from six aquafarms in different provinces of China. Furthermore, we assessed the categorization of crabs based on amino acids and fatty acids using PCA, supporting the development of a potential geographical indication label for *E. sinensis*. This study will provide a scientific basis for optimizing farming practices of *E. sinensis* and offer a reference for consumers in choosing crab products.

## 2. Materials and Methods

### 2.1. Sample Collection

A total of thirty samples (♀:♂ = 1:1) of sexually mature and physically healthy individuals were collected from each aquafarm located in the primary crab-producing regions of China. These areas included locations in Panjin, Liaoning (122.08° N, 41.00° E; PJ), Changzhou, Jiangsu (120.04° N, 31.00° E; CZ), Jinxian, Jiangxi (119.78° N, 31.80° E; JX), Yongyan, Anhui (118.13° N, 33.05° E; YY), Huzhou, Zhejiang (116.29° N, 28.38° E; HZ), and Ezhou, Hubei (114.73° N, 30.35° E; EZ). The information about the origin, body weight, and carapace length of the samples under investigation is detailed in Table 1. The sampling of the crabs in the PJ group occurred in September, while the CZ, EZ, and YY groups were sampled in October, and the HZ and JX populations were sampled in November. All samples were collected during the sales season, when sexual maturity was high in the relevant district, to ensure a consistent developmental stage for our study.

The sampled individuals were anesthetized via ice bath. Subsequently, their morphometric attributes were measured, and they were dissected to collect samples of the hepatopancreas, gonad, and muscle. All tissues were stored separately at −80 °C for further biochemical analysis. Before conducting biochemical analysis, five samples of each tissue from the same sex were mixed together to form one replicate. In total, three replicates of each tissue from the same sex were prepared. Each mixed sample was then ground into a pulp separately.

### 2.2. Measurements of Morphometric Attributes

**Figure 1:**
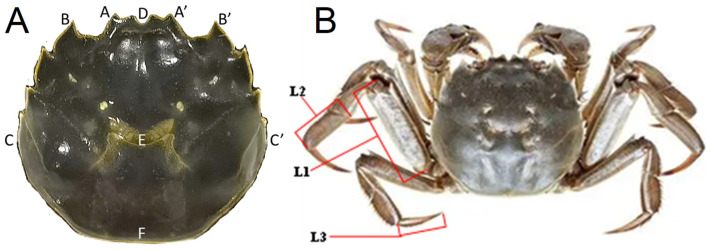
Distance measures applied to the back of carapace (**A**) and ambulatory legs of Eriocher sinensis (**B**). Definition of each measurement parameter: C1: C (C′), width of carapace; C2: DF, length of carapace; C3: A (A′), width of frontal; C4: B (B′), width of the first anterolateral tooth; C5: EF, length of the posterior half of carapace. L1: the longest dactylus length of the third ambulatory leg; L2: the frontier dactylus length of the third ambulatory leg; L3: the dactylus length of the last ambulatory leg.

The morphometric attributes, including carapace length, carapace width, body height, and others, were obtained using a Vernier caliper with an accuracy of 0.01 mm. The measurements adhered to the Chinese National Standard for Germplasm Identification for *Eriocheir sinensis* (GB/T 19957-2005) [32]. The measurement parameters were as follows: length of carapace (C1), width of carapace (C2), width of frontal (C3), body thickness (T), width of the first anterolateral tooth (C4), length of the posterior half of carapace (C5), the longest dactylus length of the third ambulatory leg (L1), the frontier dactylus length of the third ambulatory leg (L2), and the dactylus length of the last ambulatory leg (L3). Distance measures applied to the back of carapace and ambulatory legs of *E. sinensis* are shown in Figure 1.

### 2.3. Measurements of Proximate Composition

To determine the moisture content, the samples were dried using a vacuum freeze-dryer (FD-1A-50, Biocoll, Beijing, China) at a temperature of −50 °C until a constant weight was achieved. To determine the crude protein content, the Kjeldahl method was employed and the nitrogen–protein conversion factor was 6.25. To determine the crude lipid content, the Soxhlet extraction method was employed, utilizing petroleum ether as the solvent. The ash content was measured by burning the samples at a temperature of 550 °C.

### 2.4. Amino Acid Analysis and Assessments of Protein Quality

The amino acid content is determined after pre-treatment using the acid hydrolysis method [33]. Briefly, 0.1 g of samples were homogenized, and 6.0 mol·L^−1^ HCl was immediately added. The solvent was vacuumed and then reacted at 130 °C for 7 h. The hydrolysates were freeze-dried and dissolved in 0.02 mol·L^−1^ HCl. The solution was analyzed using a Hitachi 835-50 automatic amino acid analyzer (Hitachi 835-50, Tokyo, Japan) equipped with a column (Hitachi custom ion exchange resin no. 2619) for physiological fluid analysis. The column temperature was set to 57 °C, and the post-column reaction with ninhydrin solution equipment was maintained at 130 °C. The amino acid content was shown as the percentage of a particular amino acid to the sample wet weight (mg·g^−1^ tissue wet weight).

The essential amino acid score (EAAS) was calculated using a formula based on the report by the Food and Agriculture Organization (FAO) about amino acid requirements for adult maintenance [34], as described by Millward [35].

$$\mathrm{EAAS}=\frac{\mathrm{mg}\;\mathrm{of}\;\mathrm{essential}\;\mathrm{amino}\;\mathrm{acid}\;\mathrm{in}\;1\;g\;\mathrm{of}\;\mathrm{test}\;\mathrm{protein}}{\mathrm{mg}\;\mathrm{of}\;\mathrm{essential}\;\mathrm{amino}\;\mathrm{acid}\;\mathrm{in}\;\mathrm{reference}\;\mathrm{pattern}}\times 100\;$$

The amino acid chemical score (CS) was calculated using the method described by Seligson and Mackey [36], which compares the relative content of essential amino acids (EAAs) in sample proteins to that in standard egg protein.

### 2.5. Fatty Acids Analysis

Total lipids were extracted following the method described previously [37,38]. Briefly, mixtures of crab tissues were subjected to diethyl ether for 6 h. Next, the resultant crab lipid was mixed with a potassium hydroxide–methanol solution and incubated at 70 °C for 1 h to conduct saponification. Fatty acid methyl esters were gained using 12.5% (*w*/*v*) sulfuric acid–methanol reagent and separated using gas chromatography–mass spectrometry (Agilent Technologies, 7890A, Santa Clara, CA, USA). The separation was performed on an HP-88 (100 m × 0.25 mm × 0.20 μm) column. Peaks were identified by comparing the retention times with fatty acid methyl ester standards (Sigma-Aldrich, Shanghai, China). Quantitative analysis of each fatty acid component was carried out using the peak area normalization method.

### 2.6. Principal Component Analysis

Morphological proportion traits and amino acid content in both male and female crabs were analyzed using principal component analysis (PCA) using the PCA online tool (Omicshare, http://www.omicshare.com/tools/, accessed on 23 October 2024). The parameters were analyzed by default settings.

### 2.7. Statistical Analysis

All results were expressed as mean ± standard error (SE). The statistical tests were performed with IBM SPSS Statistics 26 software. All data and significant difference analyses in proximate chemical composition, amino acid analysis, and fatty acid analysis were conducted using one-way analysis of variance (ANOVA), multiple comparisons were handled using Tukey’s multiple comparison test, and *p* < 0.05 was regarded as statistical significance.

## 3. Results

### 3.1. The Morphological Parameters

**Figure 2:**
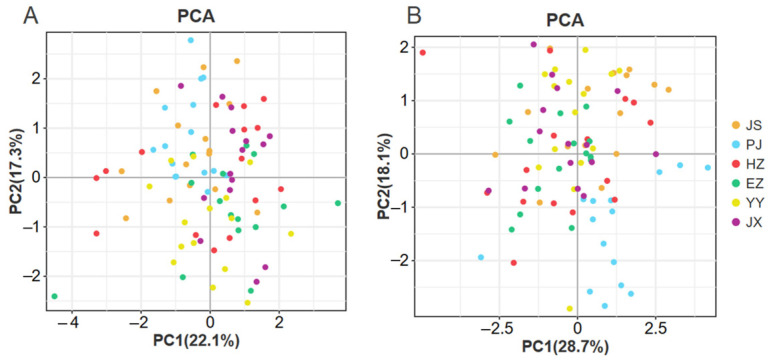
PCA analysis of male (**A**) and female (**B**) *Eriocheir sinensis* from six regions based on morphological proportion traits; YY—Yongyan in Anhui province, PJ—Panjin in Liaoning province, HZ—Huzhou in Zhejiang province, JX—Jinxian in Jiangxi province, CZ—Changzhou in Jiangsu province, EZ—Ezhou in Hubei province.

The morphological proportion traits are listed in Table 2. Among male crabs, the CZ population exhibited notably higher values for T/C1 compared to other populations, whereas the HZ population had the lowest values. Additionally, the PJ population had significantly higher values for L3/C1 than other populations, while the YY population had the smallest values. However, there were no significant differences in C2/C1 among the populations. In female crabs, the PJ population had significantly higher values for C3/C1, C4/C1, and L3/C1 than the other populations but had the lowest values for C5/C1. Conversely, there were no significant differences in the ratios of C2/C1 and L2/C1 among the populations. PCA was conducted to analyze the morphological proportion traits of male and female crabs. For male crabs, PC1 explained 22.1% of the total variance, and PC2 explained 17.3%. The cumulative contribution rate of PC1 and PC2 was 39.4%. In the results for female crabs, PC1 had a contribution rate of 28.7%, and PC2 had 18.1%. The cumulative contribution rate of PC1 and PC2 was 46.8%. According to the PCA plot (Figure 2), there was considerable overlap among the scatter points of each group, whether they were male or female, indicating that crabs from various cultured populations cannot be effectively distinguished. The overlaps indicated that certain parameters may be resistant to variations in environmental conditions or farming practices.

### 3.2. Proximate Composition

**Figure 3:**
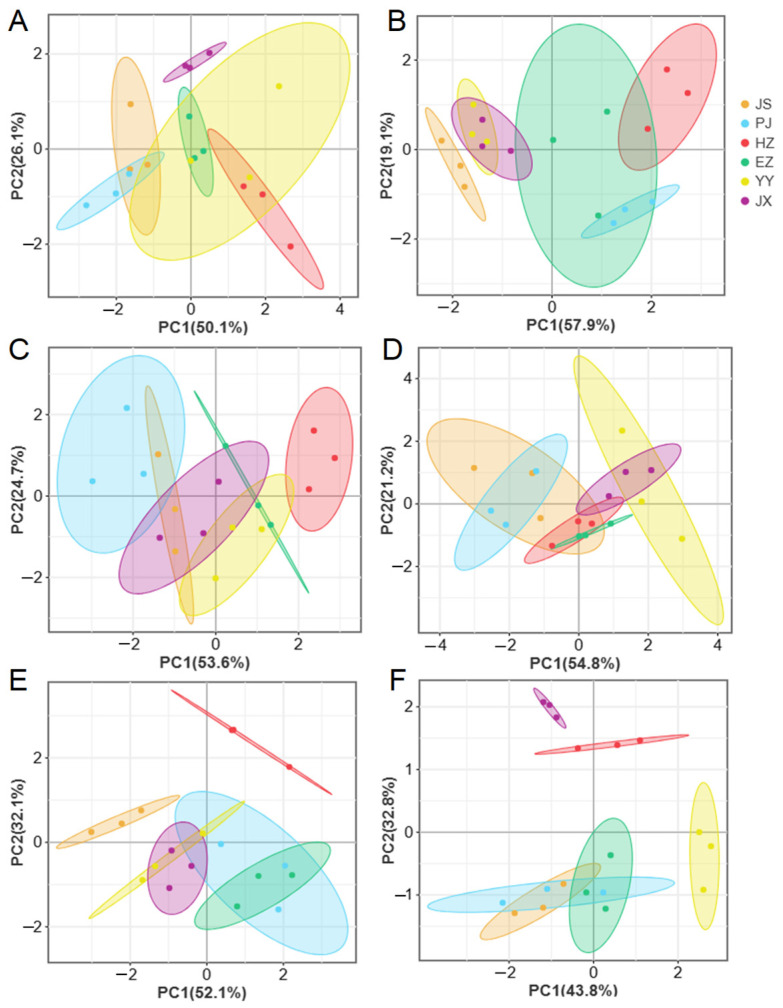
PCA analysis of male (**A**,**C**,**E**) and female (**B**,**D**,**F**) *Eriocheir sinensis* from six regions based on proximate composition of muscle (**A**,**B**), hepatopancreas (**C**,**D**), and gonad (**E**,**F**) tissues. YY—Yongyan in Anhui province, PJ—Panjin in Liaoning province, HZ—Huzhou in Zhejiang province, JX—Jinxian in Jiangxi province, CZ—Changzhou in Jiangsu province, EZ—Ezhou in Hubei province.

It is evident that there were variations in proximate composition of the edible tissues among the six populations, except for the ash content in male and female hepatopancreases (Table 3). In muscles, relatively high crude protein contents were observed in male crabs from HZ and female from YY (*p* < 0.05). Notably, the crude lipid contents in hepatopancreases were higher than other tissues, ranging from 21.73 to 41.53 g/100 g in male crabs and from 27.80 to 53.20 g/100 g in female crabs. Among male crabs, the HZ and YY groups show higher crude lipid contents compared to other groups (*p* < 0.05). Similarly, in female crabs, the YY, JX, and HZ groups exhibit higher crude lipid contents than other groups (*p* < 0.05). In female ovaries, the contents of crude proteins and crude lipids were much higher than that in testes. The highest values of crude proteins in ovaries were observed in the YY, HZ, and JX groups, while in testes, the highest values were observed in the PJ and EZ groups (*p* < 0.05). Additionally, the highest sugar contents were significantly higher in muscles and hepatopancreases from the HZ group, while in testes and ovaries, the highest values were detected in the CZ and YY groups (*p* < 0.05), respectively. The PCA revealed distinct categorizations of proximate composition in crabs from various regions (Figure 3). The cumulative contribution rates of PC1 and PC2 were from 76.0% to 84.2%, of which the contribution of PC1 was from 43.8% to 57.9%. In the muscle tissue of male crabs, distinct profiles were observed among PJ, HZ, and JX groups (Figure 3A). Similarly, in female crab muscle tissue, samples from JS, HZ, and PJ exhibited clear differentiation (Figure 3B). The male hepatopancreases showed significant variations in proximate compositions among the HZ, PJ, YY, and JX groups (Figure 3C). In testes, the proximate compositions profiles of HZ and JS could be distinct from others (Figure 3E), while the ovary profiles from HZ, JX, and YY showed clear distinctions (Figure 3F). 

### 3.3. Variation in Amino Acids Composition

Except for Tryptophane, which was not determined due to its destruction during the acid-hydrolysis process, a total of 17 amino acids were detected in the six groups of *E. sinensis* (Table 4, Table 5 and Table 6). These include seven essential amino acids (EAA), two semi-essential amino acids (SEAA), and eight non-essential amino acids (NEAA). Glu, Arg, Asp, Ala, Lys, Leu, and Gly were found to have higher contents in the three edible parts, while Cys, Met, and His had lower contents. Glu was determined to be the most abundant amino acid in the three tissues, with concentrations of 1.76–2.42 g/100 g, 0.53–1.18 g/100 g, and 1.82–3.20 g/100 g in the muscle, hepatopancreas, and gonads, respectively.

In muscles (Table 4), the levels of total amino acids (TAAs) were highest in the EZ group and lowest in the PJ group for both male and female crabs (*p* < 0.05). Similarly, the levels of EAAs were highest in the HZ group and lowest in the PJ group for both male and female crabs (*p* < 0.05). It is worth noting that in male crabs, the levels of EAAs in the HZ, EZ, YY, and CZ groups were significantly higher than in the PJ and JX groups (*p* < 0.05). In female crabs, the levels of EAAs and TAAs in the HZ, EZ, and YY groups were significantly higher than in other groups (*p* < 0.05). The EAA/TAA ratio in male crabs ranged from 0.34 to 0.36, while in female crabs, it ranged from 0.34 to 0.38. The EAA/TAA ratio in the HZ group was significantly higher than other groups in both male and female crabs (*p* < 0.05).

Table 5 shows the amino acids composition in the hepatopancreas. In male crabs, the TAA content in the EZ, HZ, and PJ groups was significantly higher than in other groups, while in female crabs, the TAA content in the HZ group was significantly higher compared to the other five groups (*p* < 0.05). In terms of EAA content, male crabs showed the highest levels in the PJ and EZ groups, while in female crabs, the HZ group had the highest EAA content. In male crabs, the EAA/TAA ratio was significantly higher in the CZ and JX groups compared to other groups (*p* < 0.05), while in female crabs, the JX group had a significantly higher EAA/TAA ratio compared to other groups (*p* < 0.05).

The amino acids composition differed significantly between the testes and ovaries, with the latter exhibiting higher amino acids content than the former. In testes, TAAs of the YY, CZ, and EZ groups was significantly higher than that of the JX groups (*p* < 0.05), while there were no significant differences among the other groups. In the testes of crabs across six groups, the YY population had the highest EAA content, while the JX group had the lowest. The EAA/TAA ratio was significantly higher in HZ compared to PJ and EZ groups (*p* < 0.05). It is worth noting that the TAA content in the ovaries was the highest compared to other tissues, with all groups exhibiting levels exceeding 22 g/100 g. The highest content was observed in YY group (24.28 g/100 g), which was significantly higher than the lowest content in the CZ group (22.32 g/100 g, *p* < 0.05), whereas no significant differences were found among the other groups. The YY and JX groups had significantly higher EAA content compared to EZ group. The EAA/TAA ratios for the JX, CZ, and HZ groups exceeded 0.40, with JX and CZ displaying significantly higher ratios compared to other groups (*p* < 0.05). The EZ group exhibited the lowest ratio among all groups.

The amino acids contents per gram of protein of the three tissues are shown in Table S1. The EAAS and CS are shown in Tables S2 and S3, respectively. The EAAS of muscles showed that the values of most amino acids were above 1 except for sulfuric amino acids (SAA, methionine + cysteine). The scoring for aromatic amino acids (AAA, phenylalanine + tyrosine) was the highest, ranging from 1.83 to 2.04. In male crab muscles, the limiting AAs in the PJ, HZ, EZ, and YY groups were SAAs, while in female crab muscles, SAAs were limited in the CZ, EZ, and HZ groups. The CS values for SAAs and valine were generally below 1 (Table S2). On the contrary, the highest CS value was observed in lysine (1.45–4.50) (Table S2). In the hepatopancreas, EAAS values of all amino acids were above 1 (Table S2), while CS values of isoleucine, leucine, and SAAs from males in the HZ, EZ, and YY groups were below 1 (Table S3). The CS of SAAs in female hepatopancreases from the HZ group was the lowest (0.70). In testes, threonine had the highest EAAS values (3.39–3.93) and CS values (2.12–2.46), while SAAs had the lowest EAAS (0.63–0.93) and CS (0.43–0.63), which were much lower than 1. The CS values of valine (0.69–0.83) in testes were also far below 1. In ovaries, the EAAS and CS of all amino acids were above 1.

**Figure 4:**
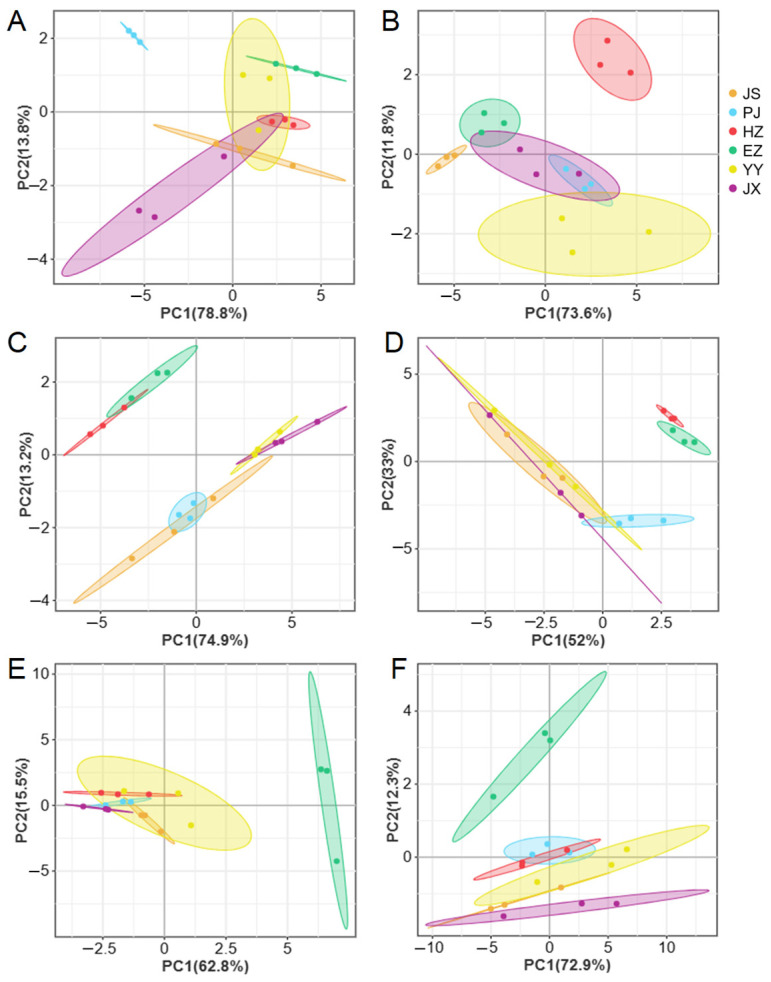
PCA analysis of male (**A**,**C**,**E**) and female (**B**,**D**,**F**) *Eriocheir sinensis* from six regions based on amino acids contents of muscle (**A**,**B**), hepatopancreas (**C**,**D**), and gonad (**E**,**F**) tissues. YY—Yongyan in Anhui province, PJ—Panjin in Liaoning province, HZ—Huzhou in Zhejiang province, JX—Jinxian in Jiangxi province, CZ—Changzhou in Jiangsu province, EZ—Ezhou in Hubei province.

The PCA revealed distinct categorizations of amino acids in crabs from various regions (Figure 4). The contribution variance rates of PC1 were from 52.0% to 78.8% in different tissues, while PC2 were from 11.8% to 33.0%. The rates of cumulative contribution of PC1 and PC2 ranged from 78.3% to 92.6%. In the muscle tissue of male crabs, distinct profiles were observed from the EZ, PJ, JS, and JX groups (Figure 4A). Similarly, in female crabs’ muscle tissue, samples from EZ, JS, HZ, and YY exhibited clear differentiation (Figure 4B). The male hepatopancreas showed significant variations in amino acid compositions among HZ, EZ, YY, and JX groups (Figure 4C). In the female hepatopancreas, EZ, PJ, and HZ could be distinguished (Figure 4D). In testes, the amino acid profiles of EZ could be distinguished from others (Figure 4E), while the ovary profiles from EZ, JX, and YY showed clear distinctions (Figure 4F).

### 3.4. Variation in Fatty Acids Profiles

The composition of fatty acids varied significantly across different regions, tissues, and genders. Specifically, this study identified 14 fatty acids in muscle tissues (Table 7), 28 in the hepatopancreas (Table 8), 14 in testes, and 22 in ovaries (Table 9).

In muscles (Table 7), four saturated fatty acids (SFAs) were identified, with C16:0 being the most abundant. Four monounsaturated fatty acids (MUFAs) were identified, with C18:1n9c being the most abundant. Additionally, six types of polyunsaturated fatty acids (PUFAs) were identified, with C20:4n6 and C18:2n6c showing the highest concentrations. In male crabs, the highest concentrations of SFAs, MUFAs, and PUFAs were recorded in the HZ group. Conversely, the lowest levels of SFAs and MUFAs were observed in the PJ group, while the lowest concentration of PUFAs was found in the EZ group. In female crabs, the SFAs, MUFAs, and PUFAs were highest in the JX group. Conversely, the lowest concentrations of SFAs were observed in the PJ group, MUFAs in the HZ group, and PUFAs in the EZ group. n-3 PUFAs were more abundant than n-6 PUFAs in the muscle tissues of both males and females, with the JX group exhibiting the highest levels.

The hepatopancreas had the richest diversity and concentration of fatty acids (Table 8). Twelve SFAs were detected, with C16:0 exhibiting the highest content. Six MUFAs were identified, with C18:1n9c being the most prevalent. Ten PUFAs were detected, with C18:2n6c having the highest concentration. In male crabs, the HZ group had the highest value of SFAs, MUFAs, and PUFAs, the CZ group had the lowest value of SFAs and MUFAs, and PUFAs were the lowest in the PJ group. The content of EPA was highest in the YY group, followed by the JX group. In female crabs, the EZ group contained the highest levels of SFAs and MUFAs, whereas the lowest were observed in the CZ group. For PUFAs, the highest content was in the YY group, with the lowest again in the CZ group. The content of n-3 PUFAs was higher in the YY group compared to other groups. The concentration of n-6 PUFAs was highest in the HZ group among males, whereas both the JX and HZ groups exhibited higher values in females.

The ovaries of female crabs contained a higher diversity and concentration of fatty acids compared to the testes of male crabs (Table 9). The testes comprised four types of SFAs, five types of MUFAs, and five types of PUFAs. The HZ population exhibited the highest concentrations of SFAs, MUFAs, and PUFAs. Conversely, the JX population had the lowest SFA content, while the PJ population had the lowest levels of MUFAs and PUFAs. In contrast, the ovaries contain ten types of SFAs, four types of MUFAs, and eight types of PUFAs, among which C16:0, C18:1n9c, and C18:2n6c were present in the highest amounts of SFAs, MUFAs, and PUFAs, respectively. The total contents of MUFAs and PUFAs were higher than that of SFAs. The highest content of n-3 and n-6 PUFAs were both in the HZ group (*p* < 0.05).

**Figure 5:**
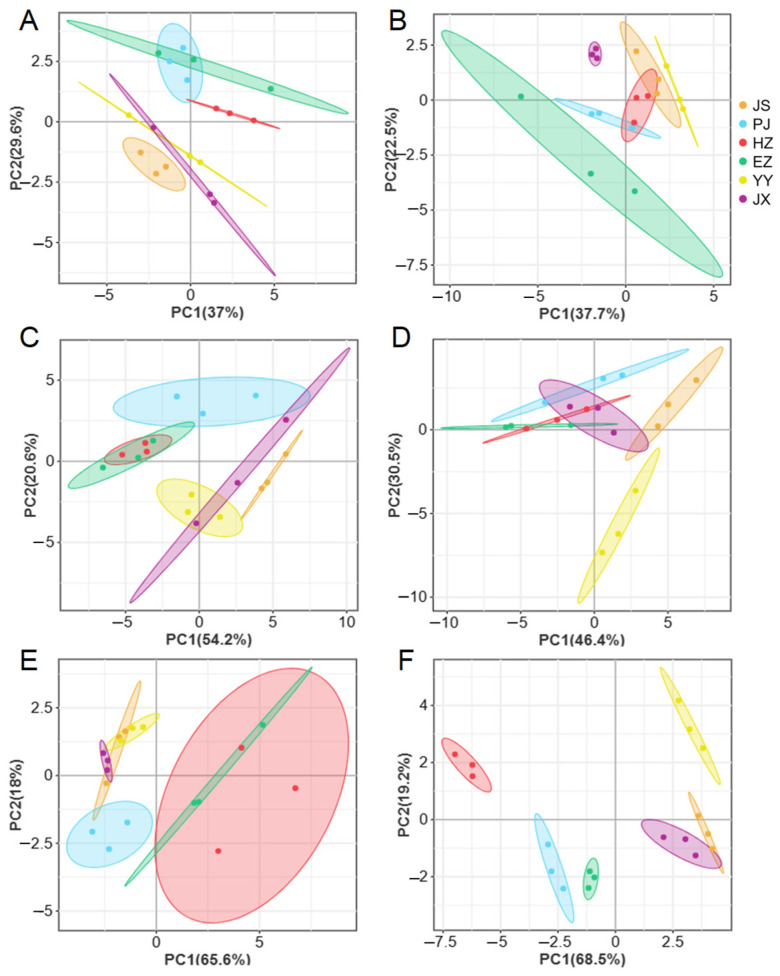
PCA analysis of male (**A**,**C**,**E**) and female (**B**,**D**,**F**) *Eriocheir sinensis* from six regions based on fatty acids contents of muscle (**A**,**B**), hepatopancreas (**C**,**D**), and gonad (**E**,**F**) tissues. YY—Yongyan in Anhui province, PJ—Panjin in Liaoning province, HZ—Huzhou in Zhejiang province, JX—Jinxian in Jiangxi province, CZ—Changzhou in Jiangsu province, EZ—Ezhou in Hubei province.

The PCA revealed distinct categorizations of fatty acids in crabs from various regions (Figure 5). The contribution variance rates of PC1 were from 37.0% to 68.5% in different tissues, while PC2 were from 18.0% to 30.5%, with cumulative contribution from 60.2% to 87.7%. Specifically, in the muscle tissue of male crabs, distinct profiles were observed for fatty acids from the JS, YY, HZ, and EZ groups (Figure 5A). Similarly, the fatty acid profiles in female crabs’ muscle tissue clearly differentiated samples from EZ, PJ, JS, JX, and YY (Figure 5B). The hepatopancreas of females showed significant variations in fatty acid compositions among PJ and JS groups (Figure 5C), while in males, distinct profiles were observed from PJ, JS, and YY (Figure 5D). In testes (Figure 5E), the fatty acid profiles of PJ were distinctly different, while the ovary profiles from all the groups showed clear distinctions (Figure 5F).

## 4. Discussion

### 4.1. The Effect of Different Regions on the Morphological Parameters

Morphological methods, which distinguish populations based on morphological differences among groups, are traditional approaches in taxonomy [39]. Reports suggested that wild crab populations from different regions could be distinct enough by morphological characteristics [28]. However, these differences did not persist in the offspring generations of artificial culture [40,41]. In our study, there were significant differences in certain measurement indicators among specific groups; for example, L3/C1 and C4/C1 in female crabs from PJ groups were significantly different from others. The discrepancies might be influenced by genetic factors, as the crabs bred in the PJ group belong to the Liaohe River system, while others come from the Yangtze River system. In addition, another reason for the morphological variation in the PJ group could be due to the higher latitude, which results in lower accumulated temperatures over the growing season [42]. Yet, PCA showed significant overlap in the scatter plots of principal components among individuals from different groups. Also, the cumulative contribution rate of PC1 and PC2 falling below 50% indicated that they were insufficient for explaining the main variability in the data, thereby failing to effectively distinguish different groups. This implies that the morphological differences among the six surveyed crab lineages were minor, complicating their distinction through traditional morphological identification methods. Our results were supported by previous studies that the morphological traits’ differences in cultured adult *E. sinensis* between various regions or selected breeding lines are relatively minor [43].

### 4.2. The Effect of Different Regions on Basic Nutrients in the Three Edible Parts

*E. sinensis* is a nutritious and flavorful freshwater food that is low in fat and calories yet high in protein. Its main edible tissues are muscles, hepatopancreases, and gonads [22]. This study revealed regional variations in ash content, with notable differences among tissues. For instance, the PJ group exhibited the highest ash content in muscle tissue, whereas the hepatopancreas showed no significant differences. This variance may be attributed to differences in culture environments, which potentially influence the transformation and absorption of inorganic salts and minerals [44]. The content of crud protein was the highest in ovaries, followed by testes and muscles, but the lowest in hepatopancreases. Similar results were also reported for *E. sinensis* reared in carbonate-alkalinity water [45]. The variability in protein content in muscle tissues, notably higher in HZ males and YY females, may suggest regional differences in available food or variations in water quality. This observation was supported by previous research indicating that environmental factors significantly influence muscle composition in aquatic species [46]. The content of crude lipids was the highest in hepatopancreases, but the lowest in muscles. The results were in accordance with previous reports [17,20]. Lipids are the major energy reserve in the hepatopancreas and can act as a source of nutrients for mobilization, aligning with former researches which associated high lipid reserves in the hepatopancreas with reproductive health and the survival strategies of crustaceans [47,48]. Regarding sexual dimorphism, female gonads exhibited higher levels of protein and fat content. This distinction is primarily due to the necessity for ovaries to accumulate substantial nutrients to support and energize embryonic development [49]. Additionally, sugar levels were highest in the reproductive tissues and lowest in the muscle tissues, supporting the concept of adaptive metabolic responses. These sugars are critical for reproductive processes such as gametogenesis, fertilization, and embryo development during the breeding season [50]. What is more, the sweet taste may be one of the factors that contributes to the popularity of testes and ovaries among consumers. Moreover, PCA revealed the distinction of *E. sinensis* from different regions on the bases of proximate composition profiles. The combined contribution rates of PC1 and PC2 were consistently equal to or greater than 76.0% in all the tissues, indicating that these two principal components were sufficient to explain the main variability, thereby effectively distinguishing different groups. Numerous studies have demonstrated that variations in the proximate composition among different groups can be attributed to differences in aquaculture conditions. Factors such as water quality [51] and microorganisms [52] have been proven to influence the biochemical compositions of aquatic animals. Wu et al. reported significant differences in the proximate composition of various tissues of *E. sinensis* when comparing wild conditions to rice farming environments [17]. Feeding practices may also contribute to variations in the proximate composition among different groups. Previous research has demonstrated that a high-quality diet can enhance the nutritional value of male *E. sinensis* [53,54]. Additionally, the maturity period significantly influences the nutritional composition of the gonads and hepatopancreases [13,15,55].

### 4.3. The Effect of Different Regions on Amino Acids Contents

Amino acids are essential not only for the physiological functions like the growth and health of *E. sinensis* but also contribute significantly to the flavor profile [56]. The type, quantity, and ratio of EAAs serve as crucial indicators of the nutritional quality of dietary proteins. According to the ideal protein pattern recommended by the FAO/WHO, a protein is considered high-quality if the ratio of EAA/TAA is approximately 0.40 and the ratio of EAA/NEAA exceeds 0.60 [57]. In this study, the proportion of EAA/TAA in muscles from six regions ranged between 0.34 and 0.38. The ratios of EAA/NEAA were above 0.60, except for female crabs in the PJ group (0.53). In the hepatopancreas, the proportions of EAA/TAA were approximately 0.40, except in the HZ and EZ groups, and the EAA/NEAA ratios were all above 0.60. Noticeably, though the crude protein content of the testes is high, the proportions of EAA/TAA were below 0.40 (ranging from 0.33 to 0.34), and the EAA/NEAA ratios were below 0.6 (ranging from 0.53 to 0.57). On the contrary, in the ovaries, the proportion of EAA/TAA was above 0.4 (0.40–0.41) and EAA/NEAA was above 0.6 (0.77–0.83). This indicates that *E. sinensis* can provide high-quality protein for human consumption, with specimens from the JX region being of higher protein quality.

The EAAS and CS are primary indicators used to compare and analyze the composition of essential amino acids in animal muscle tissue and to evaluate protein nutritional value [58]. The average EAAS of the muscles, hepatopancreases, and gonads from the six groups exceeded the requirements of the FAO standard model (>1.0), indicating that the amino acid content could meet the human nutrition standards of the human body [11,12]. The average CSs of the three tissues were approximately or above 1.0, but in testes, the CSs of Val, AAA, and Lys were below 1.0, which were identified as the limiting amino acids. It is noteworthy that the values of EAA/TAA, EAA/NEAA, EAAS, and CS all suggest that the ovary is capable of providing high-quality protein for consumption. This aligns with the fact that female crabs are indeed more sought-after among consumers and therefore command a higher market value than male crabs [59]. Additionally, these indicators suggested that the ovarian nutrition in the HZ group would be better. Moreover, the PCA uncovered that *E. sinensis* from different regions could be distinguished based on their amino acids compositions. The cumulative contribution rates of PC1 and PC2 were above 78.0%, indicating that these two principal components could explain the majority of variance. The observed variations in amino acids profiles of *E. sinensis* across different regions suggest that environmental factors or farming practices significantly influence its nutritional composition [60]. Tian et al. conducted analyses on *E. sinensis* from six distinct lakes and identified significant differences in the amino acid composition of the muscle tissue, potentially linked to geographical isolation [61]. Similarly, the free amino acid profiles in the muscles of *E. sinensis* showed significant differences between rice farming and pond farming, suggesting that cultivation methods markedly affect nutritional content [62]. Moreover, these variations in nutritional composition could be associated with different feeding practices. For instance, crabs fed a natural diet showed higher levels of EAAs in muscle compared to those given commercial feed [63].

### 4.4. The Effect of Different Regions on Fatty Acids Composition

The fatty acid composition is crucial for exploring *E.* sinensis’s physiological mechanisms and nutritional properties [6]. *E. sinensis* showed higher levels of PUFAs compared to SFAs and MUFAs in muscle tissue. Conversely, in the hepatopancreas and gonads, the levels of MUFAs were the highest. Similar to the results reported by Long et al. [64] and Ye et al. [65]. This study displayed that C16:0 was the dominant SFA, C18:1n9c was the primary MUFA, and C18:2n6c was the predominant PUFA across the muscle, hepatopancreas, and gonad tissues. These findings are consistent with previous reports on *E. sinensis* [22,65,66]. PUFAs are known for their beneficial effects on health, particularly the n-3 and n-6 fatty acids, classified as essential fatty acids [67]. The n-3 and n-6 fatty acids have various physiological functions, including cholesterol management, heart health, anti-inflammatory responses, and maintaining cell membrane integrity [68,69]. The consumption of long-chain n-3 fatty acids C20:5n3 (eicosapentaenoic acid, EPA) and C22:6n3 (docosahexaenoic acid, DHA) was recommended by several international organizations and health agencies due to their numerous health benefits [70,71]. In our study, C20:5n3 and C22:6n3 were the most abundant PUFAs in *E. sinensis*. However, the amount of n-3 and n-6 fatty acids varies among different tissues and between sexes, reflecting the distinct metabolic needs of each tissue, which is consistent with observations in the *Cancer pagurus* [72]. This study further revealed that the contents of n-3 and n-6 were highest in the hepatopancreas among the three tissues. In the hepatopancreas, the highest n-3 values were from the YY group, while the highest n-6 contents were found in males from the HZ group and females from the JX group. The major differences between sexes were detected in gonads, with ovaries having higher n-3 and n-6 contents than testes. The highest n-3 and n-6 values in ovaries were found in the HZ group. These results suggest that the n-3 and n-6 were influenced by farming regions and gender. This suggestion is supported by a previous study where the key fatty acids ARA and EPA (C20:4n6) exhibited significant differences in *E. sinensis* from four regions [62]. Meanwhile, the differences in key fatty acids between male and female *E. sinensis* have also been widely reported [73].

This study also found that the total fatty acids in the hepatopancreas were much higher compared to other tissues. High fatty acids reserves in the hepatopancreas are closely associated with the reproductive health and survival strategies of crustaceans [74]. Beyond its primary metabolic functions, the hepatopancreas also acts as a crucial energy reserve, capable of being mobilized in response to food shortages, environmental stress, or reproductive needs [75,76]. In this study, the total fatty acids in male hepatopancreases from the EZ, YY and JX groups were higher than other groups, while female hepatopancreases from the HZ, EZ and YY groups were much higher, consistent with the values of crude lipids. These data indicated that farming regions affect the fatty acid content in the hepatopancreas, further influencing its nutritional value [77].

It is noteworthy that the fatty acid compositions were more abundant and the contents significantly higher in the ovaries compared to the testes. The phenomenon could be linked to the distinct reproductive roles and needs of male and female crabs [78]. The ovaries require a larger amount of fatty acids compared to the testes to support the development and maturation of eggs, which are lipid-rich cells designed to nourish the embryo after fertilization [79,80]. The total fatty acids in the ovaries were higher in the HZ, EZ, YY, and JX groups, suggesting enhanced fertility in female *E. sinensis* from these regions. Furthermore, the PCA results revealed distinct variations in the fatty acid profiles of *E. sinensis* across multiple regional locations, highlighting the profound impact of farming environments on the nutritional components. The clear categorization of crabs based on proximate compositions, amino acids, and fatty acids using PCA underscores the possibility of developing a geographical indication label for *E. sinensis*. Similar findings have been reported in previous studies that characterized the *Procambarus clarkii* from different regions in China based on amino acids and fatty acids [81].

This study assessed the geographical variations in morphological traits and nutritional profiles of adult *E. sinensis* across six major aquaculture regions in China, generating comprehensive data valuable for evaluating the nutritional quality of *E. sinensis* from different geographic populations. However, the research also faced several limitations: (1) This study did not consider specific environmental factors and feeding practices across different farming regions, which may affect interpretations related to geographic variations in the results. Future research should incorporate external variables such as water quality, temperature, and diet to provide a more comprehensive analysis of the nutritional quality of different tissues from farmed *E. sinensis*. (2) This study focused primarily on the composition and content of amino acids and fatty acids to assess the nutritional value of *E. sinensis*. However, future research could explore other important parameters, such as mineral compositions, flavor compounds, and volatile compounds, which also contribute to nutritional value. (3) This study only collected samples from six regions. However, in China, *E. sinensis* is cultivated in 28 provinces, thus the data obtained in this study are limited. Additionally, the sampling was conducted in a specific region, influenced by local aquaculture practices and environment conditions. Therefore, the findings of this study are representative only of the particular area.

## 5. Conclusions

This study of *E. sinensis* across different aquaculture regions in China highlighted the morphological and nutritional diversity within this species. Although morphological indices did not conclusively differentiate regions via PCA, significant variations were found in proximate composition, essential amino acids, and fatty acids among the crabs from various locations. Particularly, regional variations in nutrient profiles, such as higher protein content in ovaries, predominant lipids in the hepatopancreas, and a richer presence of PUFAs in muscle tissues, were distinct. The PCA based on nutritional contents could categorize *E. sinensis* from various farmed regions. Furthermore, the EAAS and CS of amino acids, along with high levels of n-3 and n-6 PUFAs in *E. sinensis*, indicated that they were nutritionally beneficial for human consumption. In conclusion, the research offered scientific references for the nutritional evaluation of cultured *E. sinensis* in China. These regional variations in tissue-specific nutritional quality can provide valuable insights for aquaculturists into the nutritional advantages of their products. Such knowledge can prompt adjustments in dietary or breeding environments to enhance aquaculture practices. Additionally, these results can act as a significant biomarker for determining the geographical origin of aquatic products, thereby providing more comprehensive information to consumers.

## Figures and Tables

**Table 1:** The sample information of adult *Eriocheir sinensis* from six groups.

Sampling Sites	Sex	Size	Body Weight (g)	Carapace Length (mm)
Yongyan Anhui (YY)	Male	15	120.85 ± 5.98	56.99 ± 0.84
	Female	15	112.27 ± 4.06	57.52 ± 0.64
Panjin Liaoning (PJ)	Male	15	86.20 ± 1.82	50.75 ± 0.23
	Female	15	57.90 ± 2.08	45.86 ± 0.50
Huzhou Zhejiang (HZ)	Male	15	150.97 ± 1.72	61.22 ± 0.29
	Female	15	146.42 ± 2.37	63.50 ± 0.35
Jinxian Jiangxi (JX)	Male	15	246.04 ± 4.46	70.93 ± 0.27
	Female	15	166.29 ± 1.97	65.61 ± 0.37
Changzhou Jiangsu (CZ)	Male	15	212.97 ± 3.84	67.07 ± 0.49
	Female	15	140.24 ± 1.24	61.53 ± 0.24
Ezhou Hubei (EZ)	Male	15	174.12 ± 2.65	62.38 ± 0.28
	Female	15	122.89 ± 2.03	59.06 ± 0.33

**Table 2:** The morphological parameters of adult *Eriocheir sinensis* from six regions.

	HZ	PJ	EZ	YY	JX	CZ
Male						
T/C1	0.5394 ± 0.0051 ^c^	0.5442 ± 0.0052 ^bc^	0.5446 ± 0.0043 ^bc^	0.5426 ± 0.002 ^c^	0.5551 ± 0.0051 ^b^	0.5686 ± 0.0037 ^a^
C2/C1	1.1345 ± 0.0205	1.1221 ± 0.0041	1.1319 ± 0.0058	1.1337 ± 0.0035	1.1381 ± 0.0055	1.1270 ± 0.0039
C3/C1	0.2416 ± 0.0023 ^bc^	0.2442 ± 0.0025 ^c^	0.2458 ± 0.0034 ^c^	0.2426 ± 0.0035 ^c^	0.2344 ± 0.0021 ^a^	0.2328 ± 0.0018 ^a^
C4/C1	0.6347 ± 0.0029 ^a^	0.6237 ± 0.0033 ^ab^	0.6140 ± 0.0044 ^bc^	0.6097 ± 0.0039 ^c^	0.6166 ± 0.0048 ^bc^	0.6215 ± 0.0047 ^b^
C5/C1	0.5416 ± 0.0031 ^ab^	0.5370 ± 0.0023 ^b^	0.5405 ± 0.0023 ^ab^	0.5464 ± 0.0021 ^a^	0.5381 ± 0.0025 ^b^	0.5443 ± 0.0026 ^ab^
L1/C1	0.7909 ± 0.0084 ^a^	0.7973 ± 0.0043 ^a^	0.7685 ± 0.0072 ^b^	0.7893 ± 0.0073 ^a^	0.7708 ± 0.0060 ^b^	0.8022 ± 0.0061 ^a^
L2/C1	0.5544 ± 0.0078 ^ab^	0.5560 ± 0.0033 ^ab^	0.5706 ± 0.0336 ^a^	0.5519 ± 0.0057 ^ab^	0.5281 ± 0.0049 ^b^	0.5504 ± 0.0049 ^ab^
L3/C1	0.4623 ± 0.0072 ^b^	0.5183 ± 0.0044 ^a^	0.4462 ± 0.0049 ^bc^	0.4424 ± 0.0067 ^c^	0.4457 ± 0.0064 ^bc^	0.4564 ± 0.0070 ^bc^
Female						
T/C1	0.5714 ± 0.0054 ^ab^	0.5764 ± 0.0082 ^ab^	0.5678 ± 0.0036 ^b^	0.5707 ± 0.0065 ^ab^	0.5868 ± 0.0039 ^a^	0.5828 ± 0.0057 ^ab^
C2/C1	1.1120 ± 0.0202	1.1105 ± 0.0037	1.1236 ± 0.0019	1.0503 ± 0.0711	1.1236 ± 0.0022	1.1261 ± 0.0041
C3/C1	0.2394 ± 0.0025 ^ab^	0.2467 ± 0.0033 ^a^	0.2347 ± 0.0027 ^bc^	0.2336 ± 0.0023 ^bc^	0.2377 ± 0.0033 ^b^	0.2301 ± 0.0017 ^c^
C4/C1	0.6071 ± 0.0133 ^b^	0.6382 ± 0.0040 ^a^	0.6028 ± 0.0039 ^b^	0.6053 ± 0.0042 ^b^	0.6062 ± 0.0034 ^b^	0.6171 ± 0.0031 ^b^
C5/C1	0.5343 ± 0.0028 ^bc^	0.5272 ± 0.0025 ^d^	0.5293 ± 0.0024 ^cd^	0.5426 ± 0.0022 ^a^	0.5369 ± 0.0020 ^ab^	0.5314 ± 0.0023 ^bcd^
L1/C1	0.7148 ± 0.0090 ^b^	0.7240 ± 0.0086 ^b^	0.7161 ± 0.0053 ^b^	0.7278 ± 0.0040 ^ab^	0.7141 ± 0.0060 ^b^	0.7473 ± 0.0096 ^a^
L2/C1	0.5117 ± 0.0079	0.5173 ± 0.0060	0.5056 ± 0.0051	0.5199 ± 0.0041	0.5092 ± 0.0055	0.5196 ± 0.0076
L3/C1	0.4194 ± 0.0087 ^b^	0.4854 ± 0.0088 ^a^	0.4248 ± 0.0063 ^b^	0.4251 ± 0.0043 ^b^	0.4293 ± 0.0115 ^b^	0.4259 ± 0.0051 ^b^

Note: T: body thickness, C1: length of carapace, C2: width of carapace, C3: width of frontal, C4: width of the first anterolateral tooth, C5: length of the posterior half of carapace, L1: the longest dactylus length of the third ambulatory leg, L2: the frontier dactylus length of the third ambulatory leg, L3: the dactylus length of the last ambulatory leg. The abbreviations stand for different regions. YY—Yongyan in Anhui province, PJ—Panjin in Liaoning province, HZ—Huzhou in Zhejiang province, JX—Jinxian in Jiangxi province, CZ—Changzhou in Jiangsu province, EZ—Ezhou in Hubei province. Values in the same row with different superscripts are significantly different (*p* < 0.05).

**Table 3:** Proximate composition in the muscles, hepatopancreases, and gonads of adult *Eriocheir sinensis* from six different regions (%, wet weight).

	Male						Female					
	CZ	PJ	HZ	EZ	YY	JX	CZ	PJ	HZ	EZ	YY	JX
Muscle												
Moisture	78.90 ± 0.26 ^a^	78.47 ± 0.48 ^a^	73.60 ± 0.71 ^d^	76.13 ± 0.34 ^b^	75.83 ± 0.84 ^bd^	78.23 ± 0.20 ^ab^	78.03 ± 0.12 ^a^	76.7 ± 0.76 ^ab^	74.10 ± 1.21 ^b^	77.07 ± 1.38 ^a^	75.5 ± 0.21 ^ab^	77.83 ± 0.54 ^a^
Ash	1.73 ± 0.09 ^bc^	2.20 ± 0.06 ^a^	1.97 ± 0.09 ^ab^	1.63 ± 0.07 ^c^	1.67 ± 0.09 ^bc^	1.40 ± 0.06 ^c^	1.67 ± 0.09 ^b^	2.03 ± 0.03 ^a^	1.70 ± 0.06 ^b^	1.63 ± 0.15 ^b^	1.70 ± 0.06 ^b^	1.63 ± 0.09 ^b^
Crude lipid	0.83 ± 0.09 ^b^	0.90 ± 0.10 ^b^	1.07 ± 0.12 ^ab^	0.97 ± 0.03 ^b^	1.20 ± 0.21 ^a^	1.37 ± 0.12 ^a^	0.80 ± 0.06 ^c^	0.77 ± 0.09 ^cd^	0.90 ± 0.06 ^bc^	1.03 ± 0.09 ^b^	0.67 ± 0.03 ^d^	1.50 ± 0.06 ^a^
Crude protein	16.80 ± 0.46 ^c^	16.80 ± 0.23 ^c^	21.23 ± 0.79 ^a^	19.60 ± 0.21 ^b^	18.93 ± 0.37 ^b^	17.20 ± 0.10 ^c^	18.00 ± 0.25 ^b^	17.93 ± 0.64 ^b^	18.07 ± 0.84 ^b^	17.33 ± 0.23 ^b^	21.10 ± 0.20 ^a^	17.10 ± 0.36 ^b^
Sugar	0.70 ± 0.10 ^c^	1.07 ± 0.07 ^b^	1.53 ± 0.12 ^a^	0.70 ± 0.10 ^c^	1.60 ± 0.15 ^a^	1.10 ± 0.06 ^b^	0.47 ± 0.03 ^e^	1.33 ± 0.03 ^b^	2.27 ± 0.09 ^a^	1.03 ± 0.09 ^c^	0.67 ± 0.03 ^d^	1.27 ± 0.03 ^b^
Hepatopancreas												
Moisture	55.83 ± 1.43 ^b^	64.97 ± 2.53 ^a^	44.53 ± 0.87 ^c^	45.77 ± 1.49 ^c^	46.10 ± 2.17 ^c^	55.00 ± 3.10 ^b^	54.23 ± 4.69 ^a^	58.23 ± 3.67 ^a^	43.27 ± 0.49 ^b^	43.67 ± 1.79 ^b^	35.07 ± 2.78 ^b^	39.47 ± 2.14 ^b^
Ash	1.37 ± 0.15	1.50 ± 0.06	1.47 ± 0.09	1.43 ± 0.09	1.27 ± 0.09	1.30 ± 0.12	1.30 ± 0.12	1.33 ± 0.07	1.10 ± 0.06	1.37 ± 0.03	1.10 ± 0.15	1.10 ± 0.06
Crude lipid	29.27 ± 1.58 ^bc^	21.73 ± 2.67 ^c^	41.53 ± 1.59 ^a^	37.73 ± 3.62 ^ab^	40.9 ± 2.01 ^a^	32.97 ± 3.03 ^b^	29.53 ± 5.48 ^c^	27.80 ± 3.27 ^c^	37.93 ± 3.58 ^bc^	42.90 ± 2.52 ^ab^	53.20 ± 3.58 ^a^	48.47 ± 2.34 ^ab^
Crude protein	9.45 ± 0.23 ^ab^	10.09 ± 0.37 ^a^	8.08 ± 0.03 ^c^	9.56 ± 0.21 ^ab^	9.71 ± 0.25 ^ab^	8.97 ± 0.25 ^b^	11.47 ± 0.43 ^a^	10.63 ± 0.61 ^ab^	9.09 ± 0.04 ^b^	8.92 ± 0.21 ^b^	9.24 ± 1.14 ^b^	9.63 ± 0.24 ^b^
Sugar	0.83 ± 0.03 ^c^	1.20 ± 0.21 ^c^	2.33 ± 0.03 ^a^	1.77 ± 0.07 ^b^	1.23 ± 0.03 ^c^	0.87 ± 0.07 ^c^	1.50 ± 0.10 ^b^	1.50 ± 0.06 ^b^	2.40 ± 0.15 ^a^	1.23 ± 0.15 ^b^	0.73 ± 0.03 ^c^	0.60 ± 0.06 ^c^
Gonad												
Moisture	73.43 ± 0.55 ^a^	71.03 ± 0.55 ^b^	70.8 ± 0.80 ^b^	71.87 ± 0.73 ^ab^	73.47 ± 0.20 ^a^	73.30 ± 0.25 ^a^	52.77 ± 0.52 ^a^	52.97 ± 1.57 ^a^	48.70 ± 0.98 ^b^	50.13 ± 0.62 ^b^	44.37 ± 0.29 ^d^	51.53 ± 0.12 ^ab^
Ash	2.40 ± 0.10 ^a^	2.10 ± 0.06 ^ab^	1.90 ± 0.12 ^b^	2.13 ± 0.12 ^ab^	2.33 ± 0.15 ^a^	2.27 ± 0.15 ^a^	2.17 ± 0.12 ^cd^	2.40 ± 0.12 ^bc^	1.83 ± 0.34 ^cd^	2.80 ± 0.25 ^ab^	3.20 ± 0.17 ^a^	1.57 ± 0.09 ^d^
Crude lipid	0.80 ± 0.06 ^c^	1.10 ± 0.15 ^bc^	2.17 ± 0.20 ^a^	1.23 ± 0.07 ^b^	0.93 ± 0.19 ^bc^	0.83 ± 0.09 ^bc^	14.23 ± 0.62 ^b^	15.33 ± 0.77 ^ab^	16.8 ± 0.53 ^a^	14.60 ± 0.26 ^b^	16.30 ± 0.47 ^a^	15.60 ± 0.10 ^ab^
Crude protein	16.53 ± 0.15 ^d^	21.03 ± 1.12 ^a^	17.30 ± 0.65 ^cd^	21.17 ± 0.09 ^a^	18.47 ± 0.22 ^bc^	19.13 ± 0.13 ^b^	26.23 ± 0.72 ^b^	25.67 ± 2.44 ^b^	30.23 ± 0.72 ^a^	28.67 ± 0.33 ^ab^	31.33 ± 0.27 ^a^	29.70 ± 0.17 ^a^
Sugar	4.70 ± 0.10 ^a^	3.37 ± 0.13 ^c^	4.03 ± 0.15 ^b^	3.03 ± 0.09 ^c^	3.93 ± 0.03 ^b^	3.93 ± 0.13 ^b^	3.10 ± 0.12 ^a^	3.10 ± 0.10 ^a^	2.20 ± 0.35 ^b^	2.83 ± 0.19 ^a^	3.37 ± 0.03 ^a^	1.87 ± 0.03 ^c^

Note: Values in the same row with different superscripts are significantly different (*p* < 0.05). The abbreviations stand for different regions. YY—Yongyan in Anhui province, PJ—Panjin in Liaoning province, HZ—Huzhou in Zhejiang province, JX—Jinxian in Jiangxi province, CZ—Changzhou in Jiangsu province, EZ—Ezhou in Hubei province.

**Table 4:** The amino acid composition in muscle of adult *Eriocheir sinensis* from six different regions (g/100 g, wet weight).

	Male						Female					
	CZ	PJ	HZ	EZ	YY	JX	CZ	PJ	HZ	EZ	YY	JX
Aspartic acid	1.406 ± 0.054 ^b^	1.099 ± 0.005 ^b^	1.582 ± 0.021 ^a^	1.618 ± 0.033 ^a^	1.435 ± 0.018 ^b^	1.189 ± 0.079 ^c^	1.218 ± 0.010 ^c^	1.417 ± 0.011 ^b^	1.542 ± 0.009 ^a^	1.392 ± 0.015 ^b^	1.432 ± 0.049 ^b^	1.368 ± 0.021 ^b^
Threonine *	0.668 ± 0.025 ^b^	0.506 ± 0.001 ^c^	0.760 ± 0.009 ^a^	0.750 ± 0.016 ^a^	0.687 ± 0.010 ^b^	0.558 ± 0.036 ^c^	0.596 ± 0.004 ^c^	0.677 ± 0.006 ^ab^	0.708 ± 0.004 ^a^	0.636 ± 0.007 ^c^	0.713 ± 0.032 ^a^	0.658 ± 0.014 ^b^
Serine	0.557 ± 0.021 ^c^	0.453 ± 0.002 ^d^	0.647 ± 0.008 ^ab^	0.676 ± 0.016 ^a^	0.616 ± 0.008 ^b^	0.454 ± 0.030 ^d^	0.468 ± 0.003 ^d^	0.590 ± 0.005 ^c^	0.641 ± 0.007 ^a^	0.553 ± 0.006 ^c^	0.606 ± 0.023 ^ab^	0.570 ± 0.027 ^b^
Glutamic acid	2.135 ± 0.080 ^b^	1.763 ± 0.009 ^c^	2.422 ± 0.029 ^a^	2.389 ± 0.049 ^a^	2.262 ± 0.046 ^ab^	1.790 ± 0.116 ^c^	1.805 ± 0.018 ^d^	2.286 ± 0.017 ^ab^	2.391 ± 0.016 ^a^	2.029 ± 0.022 ^c^	2.165 ± 0.082 ^bc^	2.099 ± 0.041 ^c^
Glycine	1.022 ± 0.039 ^b^	0.967 ± 0.006 ^b^	0.981 ± 0.011 ^b^	1.170 ± 0.024 ^a^	1.097 ± 0.016 ^ab^	0.866 ± 0.062 ^c^	0.864 ± 0.006 ^d^	1.060 ± 0.010 ^b^	0.904 ± 0.009 ^cd^	0.942 ± 0.010 ^c^	1.136 ± 0.042 ^a^	0.946 ± 0.015 ^c^
Alanine	1.242 ± 0.048 ^b^	1.047 ± 0.007 ^c^	1.350 ± 0.018 ^ab^	1.433 ± 0.025 ^a^	1.272 ± 0.026 ^b^	1.121 ± 0.034 ^c^	1.145 ± 0.008 ^c^	1.330 ± 0.011 ^ab^	1.311 ± 0.010 ^ab^	1.197 ± 0.009 ^bc^	1.332 ± 0.063 ^a^	1.237 ± 0.036 ^b^
Cysteine	0.103 ± 0.004 ^ab^	0.044 ± 0.003 ^d^	0.084 ± 0.003 ^bc^	0.067 ± 0.004 ^c^	0.072 ± 0.018 ^c^	0.114 ± 0.007 ^a^	0.069 ± 0.001 ^b^	0.085 ± 0.002 ^ab^	0.109 ± 0.003 ^a^	0.064 ± 0.005 ^b^	0.094 ± 0.020 ^ab^	0.098 ± 0.008 ^a^
Valine *	0.703 ± 0.027 ^a^	0.538 ± 0.004 ^b^	0.739 ± 0.010 ^a^	0.745 ± 0.013 ^a^	0.707 ± 0.012 ^a^	0.604 ± 0.043 ^b^	0.611 ± 0.003 ^b^	0.699 ± 0.005 ^b^	0.731 ± 0.006 ^a^	0.639 ± 0.006 ^c^	0.716 ± 0.022 ^a^	0.681 ± 0.009 ^b^
Methionine *	0.295 ± 0.015 ^b^	0.154 ± 0.002 ^e^	0.267 ± 0.005 ^bc^	0.229 ± 0.006 ^d^	0.258 ± 0.011 ^cd^	0.336 ± 0.014 ^a^	0.214 ± 0.001 ^cd^	0.325 ± 0.004 ^ab^	0.246 ± 0.005 ^bc^	0.155 ± 0.009 ^d^	0.339 ± 0.042 ^a^	0.275 ± 0.020 ^b^
Isoleucine *	0.640 ± 0.028 ^b^	0.501 ± 0.003 ^c^	0.706 ± 0.013 ^a^	0.637 ± 0.013 ^b^	0.644 ± 0.011 ^b^	0.543 ± 0.036 ^c^	0.560 ± 0.005 ^c^	0.660 ± 0.009 ^ab^	0.687 ± 0.007 ^a^	0.528 ± 0.003 ^c^	0.649 ± 0.021 ^b^	0.633 ± 0.012 ^b^
Leucine *	1.131 ± 0.047 ^b^	0.910 ± 0.006 ^c^	1.254 ± 0.019 ^a^	1.216 ± 0.023 ^a^	1.146 ± 0.018 ^ab^	0.948 ± 0.063 ^c^	0.987 ± 0.006 ^c^	1.181 ± 0.009 ^a^	1.210 ± 0.015 ^a^	1.020 ± 0.008 ^c^	1.160 ± 0.045 ^ab^	1.110 ± 0.016 ^b^
Tyrosine	0.575 ± 0.023 ^b^	0.408 ± 0.006 ^d^	0.624 ± 0.008 ^a^	0.636 ± 0.010 ^a^	0.556 ± 0.008 ^bc^	0.529 ± 0.008 ^c^	0.503 ± 0.003 ^c^	0.565 ± 0.008 ^ab^	0.616 ± 0.011 ^a^	0.537 ± 0.007 ^b^	0.616 ± 0.026 ^a^	0.566 ± 0.028 ^ab^
Phenylalanine *	0.626 ± 0.026 ^b^	0.477 ± 0.002 ^d^	0.694 ± 0.012 ^a^	0.650 ± 0.014 ^ab^	0.634 ± 0.008 ^b^	0.534 ± 0.030 ^c^	0.543 ± 0.003 ^c^	0.639 ± 0.008 ^ab^	0.661 ± 0.013 ^a^	0.542 ± 0.008 ^c^	0.649 ± 0.019 ^ab^	0.614 ± 0.013 ^b^
Lysine *	1.195 ± 0.047 ^a^	0.953 ± 0.007 ^b^	1.264 ± 0.017 ^a^	1.300 ± 0.022 ^a^	1.214 ± 0.025 ^a^	1.013 ± 0.061 ^b^	1.039 ± 0.008 ^c^	1.241 ± 0.009 ^ab^	1.265 ± 0.010 ^a^	1.128 ± 0.006 ^c^	1.231 ± 0.044 ^ab^	1.183 ± 0.021 ^bc^
Histidine ^&^	0.322 ± 0.026 ^a^	0.230 ± 0.009 ^b^	0.317 ± 0.003 ^a^	0.313 ± 0.011 ^ab^	0.326 ± 0.009 ^a^	0.268 ± 0.018 ^b^	0.270 ± 0.002 ^c^	0.311 ± 0.007 ^b^	0.369 ± 0.028 ^a^	0.270 ± 0.006 ^c^	0.335 ± 0.013 ^ab^	0.325 ± 0.005 ^b^
Arginine ^&^	1.424 ± 0.061 ^b^	1.178 ± 0.010 ^c^	1.458 ± 0.016 ^ab^	1.561 ± 0.033 ^a^	1.420 ± 0.024 ^b^	1.234 ± 0.075 ^c^	1.222 ± 0.009 ^d^	1.437 ± 0.005 ^b^	1.541 ± 0.020 ^a^	1.354 ± 0.011 ^c^	1.357 ± 0.043 ^c^	1.346 ± 0.019 ^c^
Proline	0.818 ± 0.034 ^ab^	0.551 ± 0.003 ^c^	0.680 ± 0.025 ^b^	0.869 ± 0.016 ^a^	0.745 ± 0.0140 ^b^	0.675 ± 0.057 ^b^	0.666 ± 0.006 ^c^	0.767 ± 0.017 ^b^	0.659 ± 0.044 ^c^	0.697 ± 0.009 ^c^	0.847 ± 0.027 ^a^	0.763 ± 0.021 ^b^
EAA	5.257 ± 0.211 ^a^	4.040 ± 0.022 ^b^	5.683 ± 0.08 ^a^	5.528 ± 0.105 ^a^	5.290 ± 0.090 ^a^	4.536 ± 0.257 ^b^	4.548 ± 0.029 ^bc^	4.269 ± 0.031 ^c^	6.000 ± 0.083 ^a^	5.841 ± 0.115 ^a^	5.616 ± 0.089 ^a^	4.804 ± 0.275 ^b^
SEAA	1.746 ± 0.087 ^ab^	1.407 ± 0.017 ^bc^	1.775 ± 0.019 ^a^	1.874 ± 0.043 ^a^	1.746 ± 0.024 ^ab^	1.502 ± 0.094 ^b^	1.492 ± 0.011 ^d^	1.749 ± 0.011 ^b^	1.911 ± 0.029 ^a^	1.624 ± 0.017 ^c^	1.692 ± 0.056 ^bc^	1.671 ± 0.022 ^bc^
NEAA	7.857 ± 0.302 ^b^	6.335 ± 0.038 ^c^	8.370 ± 0.074 ^ab^	8.860 ± 0.175 ^a^	8.055 ± 0.116 ^b^	6.738 ± 0.365 ^c^	6.737 ± 0.044 ^d^	8.099 ± 0.074 ^ab^	8.171 ± 0.083 ^a^	7.411 ± 0.073 ^c^	8.226 ± 0.280 ^a^	7.647 ± 0.183 ^bc^
TAA	14.860 ± 0.600 ^b^	11.781 ± 0.076 ^c^	15.827 ± 0.171 ^ab^	16.261 ± 0.323 ^a^	15.091 ± 0.228 ^ab^	12.775 ± 0.716 ^ab^	12.778 ± 0.084 ^b^	11.781 ± 0.076 ^c^	15.827 ± 0.171 ^ab^	16.261 ± 0.323 ^a^	15.091 ± 0.228 ^b^	12.775 ± 0.716 ^c^
EAA/TAA	0.375	0.362	0.379	0.359	0.372	0.376	0.356	0.362	0.379	0.359	0.372	0.376
EAA/NEAA	0.710	0.674	0.717	0.659	0.697	0.713	0.675	0.527	0.734	0.788	0.685	0.629

Note: * indicates essential amino acid; ^&^ indicates semi-essential amino acid. EAA: total essential amino acids; SEAA: total semi-essential amino acids; NEAA: total non-essential amino acids. The abbreviations stand for different regions. YY—Yongyan in Anhui province, PJ—Panjin in Liaoning province, HZ—Huzhou in Zhejiang province, JX—Jinxian in Jiangxi province, CZ—Changzhou in Jiangsu province, EZ—Ezhou in Hubei province. Values in the same row with different superscripts are significantly different (*p* < 0.05).

**Table 5:** The amino acid composition in hepatopancreas of adult *Eriocheir sinensis* from six different regions (g/100 g, wet weight).

	Male						Female					
	CZ	PJ	HZ	EZ	YY	JX	CZ	PJ	HZ	EZ	YY	JX
Aspartic acid	0.548 ± 0.017 ^c^	0.759 ± 0.015 ^b^	1.033 ± 0.008 ^a^	1.036 ± 0.012 ^a^	0.592 ± 0.046 ^c^	0.515 ± 0.023 ^d^	0.682 ± 0.052 ^c^	0.650 ± 0.011 ^c^	1.288 ± 0.021 ^a^	1.053 ± 0.018 ^b^	0.468 ± 0.016 ^d^	0.424 ± 0.019 ^d^
Threonine *	0.353 ± 0.012 ^c^	0.428 ± 0.010 ^b^	0.548 ± 0.002 ^a^	0.559 ± 0.008 ^a^	0.339 ± 0.019 ^c^	0.335 ± 0.019 ^c^	0.406 ± 0.027 ^c^	0.396 ± 0.006 ^c^	0.628 ± 0.012 ^a^	0.556 ± 0.012 ^b^	0.310 ± 0.008 ^d^	0.268 ± 0.015 ^d^
Serine	0.222 ± 0.006 ^c^	0.350 ± 0.011 ^b^	0.379 ± 0.001 ^ab^	0.392 ± 0.005 ^a^	0.261 ± 0.019 ^c^	0.212 ± 0.022 ^d^	0.300 ± 0.023 ^c^	0.301 ± 0.005 ^c^	0.487 ± 0.009 ^a^	0.431 ± 0.008 ^b^	0.222 ± 0.004 ^d^	0.173 ± 0.004 ^e^
Glutamic acid	0.737 ± 0.025 ^c^	0.901 ± 0.008 ^b^	1.031 ± 0.003 ^a^	0.962 ± 0.016 ^ab^	0.757 ± 0.050 ^c^	0.666 ± 0.037 ^d^	0.863 ± 0.056 ^c^	0.791 ± 0.015 ^d^	1.177 ± 0.018 ^a^	1.022 ± 0.011 ^b^	0.577 ± 0.015 ^e^	0.526 ± 0.031 ^e^
Glycine	0.377 ± 0.016 ^b^	0.411 ± 0.008 ^a^	0.425 ± 0.002 ^a^	0.450 ± 0.005 ^a^	0.350 ± 0.022 ^b^	0.369 ± 0.020 ^b^	0.430 ± 0.023 ^ab^	0.410 ± 0.004 ^b^	0.458 ± 0.008 ^a^	0.453 ± 0.012 ^a^	0.326 ± 0.006 ^c^	0.305 ± 0.019 ^c^
Alanine	0.516 ± 0.025 ^d^	0.497 ± 0.006 ^d^	0.685 ± 0.005 ^b^	0.761 ± 0.007 ^a^	0.415 ± 0.022 ^e^	0.579 ± 0.035 ^c^	0.512 ± 0.02 ^cd^	0.539 ± 0.007 ^c^	0.735 ± 0.012 ^a^	0.643 ± 0.014 ^b^	0.450 ± 0.010 ^d^	0.444 ± 0.050 ^d^
Cysteine	0.058 ± 0.005 ^bc^	0.085 ± 0.002 ^a^	0.052 ± 0.001 ^c^	0.058 ± 0.002 ^b^	0.066 ± 0.004 ^b^	0.064 ± 0.006 ^b^	0.066 ± 0.005 ^a^	0.044 ± 0.002 ^b^	0.061 ± 0.003 ^a^	0.064 ± 0.002 ^a^	0.048 ± 0.003 ^b^	0.045 ± 0.002 ^b^
Valine *	0.375 ± 0.010 ^b^	0.427 ± 0.008 ^a^	0.433 ± 0.003 ^a^	0.461 ± 0.006 ^a^	0.367 ± 0.023 ^b^	0.383 ± 0.021 ^b^	0.420 ± 0.022 ^c^	0.407 ± 0.004 ^c^	0.521 ± 0.011 ^a^	0.466 ± 0.010 ^b^	0.320 ± 0.012 ^d^	0.301 ± 0.014 ^d^
Methionine *	0.153 ± 0.009 ^b^	0.239 ± 0.011 ^a^	0.175 ± 0.007 ^b^	0.173 ± 0.004 ^b^	0.189 ± 0.008 ^b^	0.178 ± 0.014 ^b^	0.162 ± 0.010 ^ab^	0.170 ± 0.020 ^ab^	0.150 ± 0.009 ^b^	0.212 ± 0.008 ^a^	0.157 ± 0.002 ^b^	0.152 ± 0.010 ^b^
Isoleucine *	0.293 ± 0.009 ^b^	0.330 ± 0.003 ^a^	0.276 ± 0.002 ^b^	0.290 ± 0.001 ^b^	0.280 ± 0.020 ^b^	0.296 ± 0.016 ^ab^	0.337 ± 0.022 ^a^	0.328 ± 0.003 ^a^	0.341 ± 0.007 ^a^	0.291 ± 0.009 ^b^	0.241 ± 0.008 ^c^	0.23 ± 0.0120 ^c^
Leucine *	0.519 ± 0.018 ^b^	0.588 ± 0.010 ^a^	0.505 ± 0.003 ^b^	0.532 ± 0.004 ^b^	0.509 ± 0.026 ^b^	0.534 ± 0.028 ^ab^	0.585 ± 0.034 ^a^	0.569 ± 0.008 ^ab^	0.588 ± 0.013 ^a^	0.529 ± 0.015 ^b^	0.443 ± 0.013 ^c^	0.419 ± 0.021 ^c^
Tyrosine	0.267 ± 0.014 ^a^	0.287 ± 0.004 ^a^	0.240 ± 0.003 ^b^	0.259 ± 0.001 ^ab^	0.259 ± 0.009 ^ab^	0.268 ± 0.024 ^ab^	0.254 ± 0.013 ^b^	0.263 ± 0.004 ^b^	0.304 ± 0.010 ^a^	0.254 ± 0.010 ^b^	0.173 ± 0.007 ^c^	0.157 ± 0.010 ^c^
Phenylalanine*	0.327 ± 0.017 ^a^	0.336 ± 0.004 ^a^	0.224 ± 0.003 ^b^	0.246 ± 0.003 ^b^	0.317 ± 0.022 ^a^	0.332 ± 0.023 ^a^	0.364 ± 0.023 ^a^	0.344 ± 0.004 ^a^	0.278 ± 0.014 ^b^	0.236 ± 0.014 ^b^	0.265 ± 0.009 ^b^	0.255 ± 0.007 ^b^
Lysine *	0.493 ± 0.016 ^a^	0.490 ± 0.005 ^a^	0.455 ± 0.002 ^ab^	0.457 ± 0.005 ^ab^	0.437 ± 0.028 ^b^	0.476 ± 0.028 ^ab^	0.552 ± 0.033 ^a^	0.505 ± 0.008 ^ab^	0.530 ± 0.011 ^a^	0.456 ± 0.011 ^bc^	0.411 ± 0.009 ^c^	0.373 ± 0.026 ^c^
Histidine ^&^	0.167 ± 0.009 ^c^	0.213 ± 0.019 ^a^	0.208 ± 0.004 ^ab^	0.227 ± 0.004 ^a^	0.188 ± 0.010 ^bc^	0.172 ± 0.006 ^c^	0.188 ± 0.010 ^bc^	0.169 ± 0.003 ^c^	0.253 ± 0.008 ^a^	0.207 ± 0.010 ^b^	0.165 ± 0.005 ^c^	0.138 ± 0.004 ^d^
Arginine ^&^	0.398 ± 0.007 ^c^	0.526 ± 0.021 ^a^	0.487 ± 0.004 ^ab^	0.491 ± 0.006 ^ab^	0.397 ± 0.014 ^c^	0.450 ± 0.039 ^c^	0.478 ± 0.027 ^b^	0.574 ± 0.006 ^a^	0.598 ± 0.013 ^a^	0.558 ± 0.014 ^a^	0.354 ± 0.010 ^c^	0.360 ± 0.034 ^c^
Proline	0.319 ± 0.019 ^c^	0.403 ± 0.014 ^b^	0.486 ± 0.006 ^a^	0.430 ± 0.013 ^b^	0.356 ± 0.005 ^c^	0.324 ± 0.015 ^c^	0.383 ± 0.033 ^ab^	0.375 ± 0.011 ^b^	0.433 ± 0.010 ^a^	0.375 ± 0.019 ^b^	0.337 ± 0.011 ^b^	0.234 ± 0.007 ^c^
EAA	2.512 ± 0.067	2.837 ± 0.026	2.615 ± 0.017	2.718 ± 0.028	2.437 ± 0.137	2.534 ± 0.148	2.826 ± 0.168 ^a^	2.719 ± 0.036 ^a^	3.037 ± 0.070 ^a^	2.747 ± 0.076 ^a^	2.146 ± 0.059 ^b^	1.998 ± 0.098 ^b^
SEAA	0.565 ± 0.016 ^b^	0.739 ± 0.039 ^a^	0.695 ± 0.008 ^ab^	0.718 ± 0.004 ^a^	0.585 ± 0.023 ^b^	0.622 ± 0.045 ^ab^	0.666 ± 0.037 ^b^	0.743 ± 0.009 ^ab^	0.850 ± 0.018 ^a^	0.765 ± 0.024 ^ab^	0.519 ± 0.015 ^c^	0.498 ± 0.038 ^c^
NEAA	3.043 ± 0.124 ^c^	3.693 ± 0.054 ^b^	4.330 ± 0.025 ^a^	4.347 ± 0.054 ^a^	3.055 ± 0.161 ^c^	2.996 ± 0.179 ^c^	3.489 ± 0.223 ^c^	3.373 ± 0.047 ^c^	4.943 ± 0.089 ^a^	4.294 ± 0.089 ^b^	2.601 ± 0.056 ^d^	2.308 ± 0.127 ^d^
TAA	6.120 ± 0.206 ^b^	7.269 ± 0.110 ^a^	7.640 ± 0.041 ^a^	7.783 ± 0.084 ^a^	6.077 ± 0.321 ^b^	6.152 ± 0.372 ^b^	6.981 ± 0.427 ^b^	6.835 ± 0.082 ^b^	8.830 ± 0.176 ^a^	7.806 ± 0.183 ^ab^	5.266 ± 0.126 ^d^	4.804 ± 0.259 ^c^
EAA/TAA	0.411	0.390	0.342	0.349	0.401	0.412	0.405	0.398	0.344	0.352	0.408	0.416
EAA/NEAA	0.827	0.768	0.604	0.625	0.797	0.846	0.811	0.806	0.614	0.640	0.825	0.867

Note: * indicates essential amino acid; ^&^ indicates semi-essential amino acid. EAA: total essential amino acids; SEAA: total semi-essential amino acids; NEAA: total non-essential amino acids, TAA: total amino acids. Note: The abbreviations stand for different regions. YY—Yongyan in Anhui province, PJ—Panjin in Liaoning province, HZ—Huzhou in Zhejiang province, JX—Jinxian in Jiangxi province, CZ—Changzhou in Jiangsu province, EZ—Ezhou in Hubei province. YY—Yongyan in Anhui province, PJ—Panjin in Liaoning province, HZ—Huzhou in Zhejiang province, JX—Jinxian in Jiangxi province, CZ—Changzhou in Jiangsu province, EZ—Ezhou in Hubei province. Values in the same row with different superscripts are significantly different (*p* < 0.05).

**Table 6:** The amino acid composition in testes and ovaries of adult *Eriocheir sinensis* from six different regions (g/100 g, wet weight).

	Male						Female					
	CZ	PJ	HZ	EZ	YY	JX	CZ	PJ	HZ	EZ	YY	JX
Aspartic acid	1.788 ± 0.014 ^a^	1.755 ± 0.015 ^a^	1.722 ± 0.031 ^ab^	1.812 ± 0.042 ^a^	1.723 ± 0.080 ^a^	1.618 ± 0.022 ^b^	2.051 ± 0.052 ^b^	2.184 ± 0.021 ^ab^	2.156 ± 0.037 ^ab^	2.174 ± 0.038 ^ab^	2.230 ± 0.063 ^a^	2.170 ± 0.079 ^ab^
Threonine *	1.372 ± 0.005 ^a^	1.257 ± 0.023 ^b^	1.160 ± 0.028 ^c^	1.228 ± 0.032 ^bc^	1.318 ± 0.055 ^ab^	1.213 ± 0.016 ^bc^	1.321 ± 0.033 ^a^	1.365 ± 0.013 ^ab^	1.330 ± 0.022 ^ab^	1.367 ± 0.025 ^ab^	1.466 ± 0.041 ^a^	1.418 ± 0.051 ^ab^
Serine	0.708 ± 0.005 ^ab^	0.696 ± 0.015 ^ab^	0.638 ± 0.019 ^c^	0.694 ± 0.005 ^c^	0.743 ± 0.025 ^a^	0.628 ± 0.010 ^c^	1.354 ± 0.036 ^c^	1.484 ± 0.014 ^ab^	1.425 ± 0.023 ^bc^	1.455 ± 0.027 ^abc^	1.557 ± 0.041 ^a^	1.449 ± 0.051 ^bc^
Glutamic acid	1.949 ± 0.004 ^b^	1.967 ± 0.025 ^b^	2.024 ± 0.040 ^ab^	2.110 ± 0.035 ^a^	2.075 ± 0.047 ^a^	1.824 ± 0.023 ^c^	2.846 ± 0.076 ^b^	3.046 ± 0.033 ^ab^	2.970 ± 0.049 ^b^	2.946 ± 0.050 ^b^	3.200 ± 0.094 ^a^	2.993 ± 0.113 ^ab^
Glycine	0.588 ± 0.008 ^a^	0.583 ± 0.010 ^a^	0.585 ± 0.006 ^a^	0.594 ± 0.006 ^a^	0.594 ± 0.020 ^a^	0.549 ± 0.006 ^b^	1.182 ± 0.028 ^ab^	1.190 ± 0.016 ^ab^	1.159 ± 0.019 ^ab^	1.125 ± 0.019 ^b^	1.242 ± 0.035 ^a^	1.231 ± 0.047 ^a^
Alanine	1.070 ± 0.017	1.098 ± 0.034	1.036 ± 0.035	1.107 ± 0.018	1.038 ± 0.055	1.015 ± 0.013	1.310 ± 0.033	1.322 ± 0.014	1.336 ± 0.023	1.310 ± 0.024	1.391 ± 0.041	1.370 ± 0.050
Cysteine	0.288 ± 0.015 ^a^	0.229 ± 0.003 ^b^	0.192 ± 0.002 ^b^	0.236 ± 0.009 ^b^	0.232 ± 0.034 ^b^	0.234 ± 0.004 ^b^	0.226 ± 0.005 ^b^	0.224 ± 0.005 ^b^	0.254 ± 0.005 ^b^	0.236 ± 0.001 ^ab^	0.246 ± 0.007 ^a^	0.240 ± 0.010 ^ab^
Valine *	0.518 ± 0.001 ^b^	0.496 ± 0.013 ^b^	0.565 ± 0.005 ^a^	0.575 ± 0.019 ^a^	0.592 ± 0.027 ^a^	0.494 ± 0.007 ^b^	1.481 ± 0.035 ^ab^	1.496 ± 0.015 ^ab^	1.472 ± 0.023 ^b^	1.444 ± 0.025 ^b^	1.589 ± 0.045 ^a^	1.554 ± 0.058 ^ab^
Methionine *	0.026 ± 0.016 ^ab^	0.028 ± 0.003 ^b^	0.040 ± 0.006 ^a^	0.019 ± 0.002 ^b^	0.048 ± 0.008 ^a^	0.012 ± 0.001 ^b^	0.585 ± 0.012 ^b^	0.441 ± 0.004 ^c^	0.564 ± 0.009 ^c^	0.407 ± 0.007 ^c^	0.601 ± 0.015 ^b^	0.743 ± 0.032 ^a^
Isoleucine *	0.703 ± 0.007 ^ab^	0.650 ± 0.002 ^b^	0.617 ± 0.014 ^b^	0.730 ± 0.035 ^a^	0.680 ± 0.033 ^ab^	0.620 ± 0.010 ^b^	1.088 ± 0.026 ^ab^	1.107 ± 0.013 ^ab^	1.098 ± 0.014 ^b^	1.034 ± 0.025 ^b^	1.133 ± 0.033 ^a^	1.127 ± 0.039 ^a^
Leucine *	1.018 ± 0.016 ^ab^	0.923 ± 0.012 ^c^	0.915 ± 0.023 ^c^	1.046 ± 0.035 ^a^	1.005 ± 0.050 ^ab^	0.934 ± 0.012 ^bc^	1.886 ± 0.045	1.945 ± 0.020	1.924 ± 0.031	1.878 ± 0.042	2.001 ± 0.056	1.990 ± 0.072
Tyrosine	0.402 ± 0.018 ^a^	0.343 ± 0.003 ^b^	0.373 ± 0.009 ^ab^	0.387 ± 0.015 ^ab^	0.382 ± 0.026 ^ab^	0.364 ± 0.007 ^ab^	1.005 ± 0.026	1.025 ± 0.009	1.053 ± 0.011	1.094 ± 0.049	1.046 ± 0.033	1.051 ± 0.034
Phenylalanine *	0.564 ± 0.018 ^a^	0.508 ± 0.004 ^ab^	0.504 ± 0.017 ^ab^	0.480 ± 0.019 ^b^	0.540 ± 0.042 ^ab^	0.517 ± 0.012 ^ab^	1.133 ± 0.033	1.159 ± 0.010	1.120 ± 0.010	1.200 ± 0.077	1.190 ± 0.036	1.168 ± 0.039
Lysine *	0.678 ± 0.006 ^cd^	0.690 ± 0.020 ^cd^	0.829 ± 0.015 ^a^	0.730 ± 0.026 ^bc^	0.760 ± 0.038 ^b^	0.653 ± 0.011 ^d^	1.585 ± 0.037	1.668 ± 0.021	1.637 ± 0.030	1.617 ± 0.028	1.703 ± 0.051	1.669 ± 0.061
Histidine ^&^	0.339 ± 0.001 ^b^	0.327 ± 0.013 ^b^	0.327 ± 0.014 ^b^	0.333 ± 0.006 ^b^	0.400 ± 0.012 ^a^	0.312 ± 0.004 ^b^	0.518 ± 0.013 ^b^	0.544 ± 0.015 ^ab^	0.508 ± 0.006 ^b^	0.454 ± 0.010 ^c^	0.572 ± 0.016 ^a^	0.545 ± 0.016 ^ab^
Arginine ^&^	0.532 ± 0.007 ^b^	0.541 ± 0.013 ^b^	0.619 ± 0.021 ^a^	0.531 ± 0.014 ^b^	0.620 ± 0.025 ^a^	0.534 ± 0.009 ^b^	1.634 ± 0.035 ^b^	1.755 ± 0.013 ^ab^	1.646 ± 0.028 ^b^	1.653 ± 0.018 ^b^	1.804 ± 0.054 ^a^	1.743 ± 0.064 ^ab^
Proline	2.078 ± 0.035 ^a^	1.871 ± 0.009 ^b^	1.524 ± 0.065 ^c^	1.900 ± 0.021 ^ab^	1.960 ± 0.126 ^ab^	1.755 ± 0.030 ^b^	1.113 ± 0.029 ^bc^	1.160 ± 0.038 ^bc^	1.095 ± 0.049 ^c^	1.233 ± 0.034 ^b^	1.309 ± 0.053 ^a^	1.184 ± 0.034 ^bc^
EAA	4.879 ± 0.048 ^ab^	4.551 ± 0.069 ^cd^	4.629 ± 0.095 ^bcd^	4.808 ± 0.075 ^abc^	4.942 ± 0.172 ^a^	4.443 ± 0.068 ^d^	9.079 ± 0.22 ^ab^	9.181 ± 0.093 ^ab^	9.145 ± 0.139 ^ab^	8.948 ± 0.229 ^b^	9.683 ± 0.275 ^a^	9.670 ± 0.351 ^a^
SEAA	0.872 ± 0.006 ^c^	0.868 ± 0.022 ^c^	0.947 ± 0.035 ^b^	0.863 ± 0.008 ^c^	1.019 ± 0.036 ^a^	0.846 ± 0.012 ^c^	2.152 ± 0.047 ^bc^	2.299 ± 0.028 ^ab^	2.153 ± 0.034 ^bc^	2.107 ± 0.028 ^c^	2.376 ± 0.069 ^a^	2.288 ± 0.080 ^ab^
NEAA	8.872 ± 0.097 ^a^	8.542 ± 0.057 ^ab^	8.094 ± 0.206 ^b^	8.84 ± 0.066 ^a^	8.746 ± 0.396 ^a^	7.987 ± 0.113 ^b^	11.086 ± 0.279 ^b^	11.634 ± 0.147 ^ab^	11.449 ± 0.216 ^ab^	11.572 ± 0.202 ^b^	12.222 ± 0.363 ^a^	11.688 ± 0.418 ^ab^
TAA	14.623 ± 0.149 ^a^	13.962 ± 0.136 ^abc^	13.669 ± 0.335 ^bc^	14.511 ± 0.131	14.707 ± 0.564 ^a^	13.277 ± 0.193 ^c^	22.317 ± 0.546 ^b^	23.114 ± 0.268 ^ab^	22.747 ± 0.388 ^ab^	22.627 ± 0.458 ^b^	24.28 ± 0.706 ^a^	23.647 ± 0.849 ^ab^
EAA/TAA	0.334	0.326	0.339	0.331	0.336	0.335	0.407	0.397	0.402	0.395	0.399	0.409
EAA/NEAA	0.550	0.533	0.572	0.544	0.566	0.556	0.819	0.789	0.799	0.773	0.792	0.827

Note: * indicates essential amino acid; ^&^ indicates semi-essential amino acid. EAA: total essential amino acids; SEAA: total semi-essential amino acids; NEAA: total non-essential amino acids. The abbreviations stand for different regions. YY—Yongyan in Anhui province, PJ—Panjin in Liaoning province, HZ—Huzhou in Zhejiang province, JX—Jinxian in Jiangxi province, CZ—Changzhou in Jiangsu province, EZ—Ezhou in Hubei province. Values in the same row with different superscripts are significantly different (*p* < 0.05).

**Table 7:** The fatty acid composition in muscles of adult *Eriocheir sinensis* from different regions (mg/100 g wet weight).

	Male						Female					
	CZ	PJ	HZ	EZ	YY	JX	CZ	PJ	HZ	EZ	YY	JX
C16:0	95.95 ± 2.30	80.94 ± 3.28	104.46 ± 6.44	86.5 ± 13.37	89.89 ± 9.32	103.70 ± 12.36	93.01 ± 3.96	83.07 ± 5.07	82.15 ± 1.32	97.54 ± 18.96	93.52 ± 6.89	105.23 ± 1.46
C18:0	74.4 ± 1.32 ^ab^	59.13 ± 4.01 ^bc^	82.44 ± 3.58 ^a^	56.71 ± 8.01 ^c^	68.84 ± 2.9 ^abc^	71.83 ± 8.36 ^abc^	76.60 ± 3.16 ^a^	66.85 ± 1.85 ^ab^	74.6 ± 1.52 ^a^	57.96 ± 8.00 ^b^	66.36 ± 2.24 ^ab^	72.57 ± 2.37 ^a^
C20:0	8.37 ± 0.11 ^b^	8.01 ± 0.25 ^b^	8.55 ± 0.26 ^b^	10.38 ± 1.1 ^a^	7.45 ± 0.64 ^b^	7.79 ± 0.18 ^b^	8.49 ± 0.14 ^b^	7.91 ± 0.48 ^b^	7.46 ± 0.23 ^b^	10.83 ± 0.72 ^a^	7.89 ± 0.24 ^b^	7.54 ± 0.22 ^b^
C22:0	7.33 ± 0.04 ^ab^	6.75 ± 0.11 ^b^	8.01 ± 0.25 ^ab^	9.56 ± 1.38 ^a^	8.71 ± 0.96 ^ab^	8.56 ± 0.94 ^ab^	7.95 ± 0.17 ^b^	7.51 ± 0.35 ^b^	7.38 ± 0.26 ^b^	10.23 ± 0.77 ^a^	7.49 ± 0.23 ^b^	8.19 ± 0.52 ^b^
ΣSFA	186.04 ± 3.33 ^ab^	154.33 ± 6.25 ^b^	203.47 ± 10.52 ^a^	163.15 ± 23.69 ^ab^	174.89 ± 11.6 ^ab^	191.89 ± 21.56 ^ab^	186.04 ± 6.55	165.34 ± 2.42	171.59 ± 2.80	176.56 ± 27.81	175.27 ± 8.60	193.53 ± 3.11
C16:1	14.01 ± 0.63 ^b^	10.89 ± 0.13 ^b^	22.67 ± 3.65 ^ab^	25.65 ± 3.44 ^a^	17.16 ± 4.00 ^ab^	18.67 ± 3.05 ^ab^	17.51 ± 1.61 ^b^	17.08 ± 1.73 ^b^	16.63 ± 3.18 ^b^	31.06 ± 8.88 ^a^	20.99 ± 1.70 ^ab^	23.72 ± 0.49 ^ab^
C18:1n9c	133.92 ± 4.68 ^bc^	107.51 ± 3.38 ^c^	174.35 ± 4.94 ^a^	125.13 ± 20.73 ^bc^	140.68 ± 13.05 ^abc^	157.03 ± 17.92 ^ab^	146.92 ± 4.27 ^ab^	127.29 ± 2.68 ^b^	132.17 ± 8.17 ^ab^	144.33 ± 31.89 ^ab^	132.31 ± 5.19 ^ab^	169.78 ± 1.39 ^a^
C20:1	12.60 ± 0.62 ^a^	4.26 ± 0.11 ^c^	5.11 ± 0.42 ^c^	5.76 ± 1.01 ^c^	9.04 ± 1.05 ^b^	10.89 ± 1.31 ^ab^	10.15 ± 0.40 ^b^	4.64 ± 0.22 ^d^	5.15 ± 0.45 ^d^	5.41 ± 0.76 ^d^	14.56 ± 0.30 ^a^	7.19 ± 0.48 ^c^
C22:1n9	12.36 ± 0.74 ^cd^	18.33 ± 1.84 ^ab^	19.55 ± 0.72 ^a^	15.52 ± 0.83 ^bc^	11.46 ± 1.41 ^d^	15.57 ± 0.71 ^bc^	11.91 ± 1.50 ^ab^	16.15 ± 2.15 ^ab^	9.91 ± 2.17 ^b^	17.29 ± 2.58 ^a^	11.06 ± 1.04 ^ab^	13.20 ± 2.55 ^ab^
ΣMUFA	172.89 ± 6.10 ^bc^	141.00 ± 1.75 ^c^	221.67 ± 3.52 ^a^	172.06 ± 25.69 ^bc^	178.34 ± 18.23 ^abc^	202.15 ± 19.69 ^ab^	186.48 ± 6.35	165.15 ± 4.62	163.86 ± 12.94	198.09 ± 40.55	178.91 ± 4.85	213.89 ± 3.73
C18:2n6c	39.02 ± 5.79 ^d^	88.61 ± 15.11 ^ab^	112.55 ± 5.34 ^a^	74.66 ± 13.25 ^bc^	56.39 ± 9.50 ^cd^	53.77 ± 8.25 ^cd^	35.06 ± 2.80 ^b^	94.06 ± 15.22 ^a^	70.58 ± 2.27 ^a^	73.39 ± 11.25 ^a^	26.35 ± 3.55 ^b^	90.8 ± 6.66 ^a^
C18:3n3	5.09 ± 0.80 ^b^	13.45 ± 3.16 ^a^	11.01 ± 2.88 ^ab^	8.97 ± 1.13 a^b^	7.10 ± 1.94 ^b^	5.99 ± 0.79 ^b^	5.27 ± 0.76 ^d^	13.26 ± 0.57 ^ab^	11.00 ± 0.99 ^b^	14.21 ± 0.91 ^a^	5.87 ± 0.33 ^d^	7.79 ± 0.30 ^c^
C20:2	7.52 ± 0.61 ^c^	13.59 ± 1.06 ^ab^	15.49 ± 0.24 ^a^	16.77 ± 2.95 ^a^	10.62 ± 1.29 ^bc^	10.85 ± 1.26 ^bc^	9.79 ± 0.70 ^b^	13.13 ± 1.68 ^ab^	10.99 ± 0.38 ^ab^	13.18 ± 2.73 ^ab^	5.99 ± 0.78 ^c^	14.52 ± 0.56 ^a^
C20:4n6	17.77 ± 2.70 ^c^	38.72 ± 2.89 ^ab^	38.86 ± 1.00 ^ab^	48.15 ± 6.92 ^a^	26.03 ± 6.07 ^b^	26.54 ± 2.49 ^b^	19.45 ± 2.15 ^c^	34.12 ± 5.78 ^a^	20.93 ± 2.54 ^bc^	31.03 ± 7.15 ^ab^	14.48 ± 1.36 ^c^	32.16 ± 0.7 ^ab^
C20:5n3	56.8 ± 10.60 ^b^	75.58 ± 6.12 ^ab^	85.18 ± 2.38 ^ab^	52.14 ± 7.57 ^b^	75.47 ± 23.02 ^ab^	101.5 ± 13.81 ^a^	73.50 ± 8.20 ^a^	71.28 ± 3.95 ^a^	42.27 ± 3.60 ^b^	41.09 ± 5.81 ^b^	58.35 ± 7.47 ^ab^	83.44 ± 3.46 ^a^
C22:6n3	75.44 ± 17.21 ^b^	51.05 ± 7.14 b^c^	74.92 ± 5.01 ^bc^	30.42 ± 4.59 ^c^	78.91 ± 25.82 ^ab^	121.97 ± 14.16 ^a^	92.63 ± 11.72 ^a^	44.14 ± 6.52 ^b^	50.11 ± 7.01 ^b^	26.68 ± 3.41 ^b^	44.08 ± 13.23 ^b^	93.31 ± 7.11 ^a^
ΣPUFA	201.64 ± 35.96 ^bc^	281.00 ± 23.58 ^ab^	338 ± 16.49 ^a^	231.11 ± 36.11 ^ab^	254.51 ± 65.29 ^ab^	320.63 ± 38.28 ^ab^	235.7 ± 24.91 ^a^	269.99 ± 31.60 ^ab^	205.89 ± 10.24 ^b^	199.58 ± 30.35 ^b^	165.12 ± 18.12 ^b^	322.03 ± 4.05 ^a^
Σn-3 PUFA	137.32 ± 27.12 ^b^	140.08 ± 11.07 ^b^	171.11 ± 10.09 ^ab^	91.53 ± 13.11 ^b^	161.47 ± 50.06 ^ab^	229.46 ± 26.76 ^a^	171.4 ± 19.8 ^a^	128.68 ± 10.32 ^ab^	103.38 ± 9.63 ^b^	81.98 ± 9.38 ^b^	118.3 ± 13.94 ^b^	184.54 ± 10.01 ^a^
Σn-6 PUFA	56.79 ± 8.39 ^c^	127.33 ± 16.87 ^a^	151.4 ± 6.30 ^a^	122.82 ± 20.06 ^ab^	82.42 ± 14.34 ^bc^	80.31 ± 10.62 ^c^	51.17 ± 6.58 ^b^	128.18 ± 20.42 ^a^	91.51 ± 0.46 ^a^	104.42 ± 18.31 ^a^	40.83 ± 4.73 ^b^	122.96 ± 7.36 ^a^

Note: SFA: Saturated fatty acid; MUFA: Monounsaturated fatty acid; PUFA: Polyunsaturated fatty acid. The abbreviations stand for different regions. YY—Yongyan in Anhui province, PJ—Panjin in Liaoning province, HZ—Huzhou in Zhejiang province, JX—Jinxian in Jiangxi province, CZ—Changzhou in Jiangsu province, EZ—Ezhou in Hubei province. Values in the same row with different superscripts are significantly different (*p* < 0.05).

**Table 8:** The fatty acid composition in hepatopancreases of adult *Eriocheir sinensis* from different regions (mg/100 g wet weight).

	Male						Female					
	CZ	PJ	HZ	EZ	YY	JX	CZ	PJ	HZ	EZ	YY	JX
C12:0	23.56 ± 2.54 ^c^	31.77 ± 14.08 ^c^	79.41 ± 5.13 ^a^	56.11 ± 1.47 ^b^	38.49 ± 2.23 ^bc^	29.16 ± 5.91 ^c^	22.26 ± 0.52 ^c^	69.90 ± 12.26 ^b^	110.95 ± 12.64 ^a^	60.06 ± 6.06 ^b^	46.55 ± 3.57 ^bc^	53.01 ± 4.20 ^b^
C13:0	11.34 ± 0.78 ^b^	18.47 ± 7.71 ^ab^	28.78 ± 2. 07 ^a^	29.03 ± 1.96 ^a^	20.19 ± 0.63 ^ab^	15.49 ± 3.34 ^b^	10.08 ± 0.66 ^b^	34.10 ± 5.87 ^a^	29.94 ± 3.03 ^a^	31.04 ± 4.25 ^a^	26.85 ± 2.76 ^a^	26.27 ± 5.58 ^a^
C14:0	489.17 ± 62.06 ^bc^	205.24 ± 31.38 ^c^	510.42 ± 75.82 ^b^	532.89 ± 63.9 ^ab^	816.00 ± 54.53 ^a^	602.27 ± 194.98 ^ab^	332.39 ± 76.84 ^bc^	263.31 ± 30.05 ^c^	571.52 ± 8.93 ^b^	591.07 ± 12.27 ^b^	1569.32 ± 171.52 ^a^	540.97 ± 83.38 ^b^
C15:0	127.79 ± 14.5 ^c^	174.56 ± 25.61 ^bc^	202.79 ± 10.56 ^b^	301.82 ± 13.36 ^a^	209.16 ± 9.14 ^b^	150.09 ± 40.93 ^b^	119.65 ± 24.74 ^c^	186.23 ± 32.68 ^b^	220.26 ± 10.59 ^b^	301.60 ± 21.03 ^a^	320.76 ± 26.56 ^a^	207.48 ± 7.75 ^b^
C16:0	4071.8 ± 628.5 ^c^	4706.5 ± 605.1 ^c^	9518.7 ± 759.1 ^a^	7710.6 ± 955.6 ^ab^	6450.8 ± 281.2 ^bc^	5381.6 ± 1592.3 ^bc^	5263.7 ± 1201.4 ^c^	5635.6 ± 747.3 ^bc^	8212.2 ± 643.9 ^a^	9282.2 ± 373.8 ^a^	8256.9 ± 561.9 ^a^	7673.3 ± 233.6 ^ab^
C17:0	105.95 ± 11.10 ^c^	139.48 ± 28.32 ^b^	191.88 ± 9.82 ^ab^	241.02 ± 15.94 ^a^	153.88 ± 10.32 ^bc^	135.09 ± 32.46 ^bc^	103.6 ± 23.21 ^c^	167.45 ± 25.52 ^bc^	188.33 ± 15.36 ^b^	259.54 ± 29.39 ^a^	201.02 ± 11.27 ^ab^	163.61 ± 14.56 ^bc^
C18:0	621.77 ± 72.56 ^c^	824.66 ± 152.2 ^c^	1532.31 ± 128.38 ^a^	1275.07 ± 93.06 ^ab^	947.54 ± 41.94 ^bc^	852.47 ± 225.25 ^c^	733.35 ± 83.95 ^c^	1219.5 ± 214.78 ^ab^	1441.05 ± 110.24 ^ab^	1590.57 ± 134.6 ^a^	1152.79 ± 31.26 ^b^	1233.58 ± 64.22 ^ab^
C20:0	79.26 ± 6.04 ^d^	102.75 ± 11.81 ^bcd^	162.80 ± 5.70 ^a^	133.74 ± 10.5 ^abc^	136.05 ± 3.45 ^ab^	96.50 ± 23.59 ^cd^	64.97 ± 8.55 ^b^	145.45 ± 29.70 ^a^	150.16 ± 12.10 ^a^	161.98 ± 29.92 ^a^	168.83 ± 18.31 ^a^	127.92 ± 8.02 ^a^
C21:0	19.70 ± 1.09 ^c^	46.03 ± 5.47 ^b^	70.35 ± 4.83 ^a^	66.47 ± 1.06 ^a^	40.93 ± 4.55 ^b^	24.74 ± 5.18 ^c^	22.95 ± 3.98 ^c^	53.08 ± 11.08 ^ab^	76.25 ± 12.12 ^a^	72.62 ± 10.43 ^a^	38.48 ± 2.38 ^bc^	43.56 ± 0.54 ^bc^
C22:0	52.20 ± 4.75 ^c^	130.80 ± 32.48 ^ab^	154.05 ± 14.89 ^a^	98.99 ± 10.15 ^bc^	86.90 ± 3.71 ^bc^	69.05 ± 13.61 ^c^	50.95 ± 7.33 ^c^	135.97 ± 22.20 ^a^	142.76 ± 11.99 ^a^	131.69 ± 29.05 ^a^	75.20 ± 2.08 ^bc^	108.01 ± 15 ^ab^
C23:0	26.96 ± 1.72 ^d^	87.99 ± 20.32 ^ab^	98.29 ± 5.97 ^a^	58.68 ± 5.69 ^bc^	46.37 ± 3.78 ^cd^	34.27 ± 6.706 ^cd^	22.93 ± 2.60 ^c^	88.84 ± 20.26 ^a^	98.67 ± 6.36 ^a^	78.31 ± 17.41 ^ab^	30.27 ± 1.74 ^c^	55.33 ± 6.41 ^bc^
C24:0	65.27 ± 5.81 ^c^	106.19 ± 23.38 ^bc^	121.54 ± 3.95 ^b^	173.88 ± 8.25 ^a^	119.51 ± 11.23 ^b^	86.75 ± 26.72 ^bc^	48.34 ± 8.60 ^c^	141.64 ± 18.4 ^ab^	120.49 ± 10.34 ^ab^	170.10 ± 31.79 ^a^	107.02 ± 6.10 ^b^	92.54 ± 15.09 ^bc^
ΣSFA	5694.7 ± 796.1 ^c^	6574.4 ± 850.1 ^c^	12,671.3 ± 952.7 ^a^	10,678.3 ± 1153.9 ^ab^	9065.8 ± 301.4 ^abc^	7477.4 ± 2160.6 ^bc^	6795.2 ± 1434.1 ^c^	8141.1 ± 1137.6 ^bc^	11,362.6 ± 836.7 ^a^	12,730.8 ± 683.8 ^a^	11,994.1 ± 794.9 ^a^	10,325.6 ± 322.9 ^ab^
C14:1	43.61 ± 13.48 b^c^	22.86 ± 4.99 ^c^	68.63 ± 16.21 ^ab^	102.20 ± 10.69 ^a^	89.36 ± 6.59 ^a^	63.51 ± 23.88 ^abc^	29.00 ± 0.91 ^d^	51.87 ± 12.17 ^cd^	83.28 ± 6.59 ^b^	100.41 ± 8.09 ^b^	163.79 ± 17.31 ^a^	70.25 ± 4.94 ^bc^
C16:1	1268.3 ± 332.8 ^c^	1279.8 ± 261.1 ^c^	3391.9 ± 723.8 ^ab^	4030.9 ± 554.2 ^a^	2662.5 ± 142.4 ^abc^	1887.2 ± 669.7 ^bc^	1671.6 ± 92.4 ^c^	1819.1 ± 284.8 ^c^	2800.8 ± 292.6 ^b^	3919.3 ± 304.5 ^a^	3311.7 ± 377.7 ^ab^	2781.1 ± 155.5 ^b^
C18:1n9c	4115.4 ± 641.1 ^c^	6718.9 ± 762.9 ^c^	11,908.9 ± 559.6 ^a^	10,118.2 ± 1072.2 ^ab^	7179.1 ± 330.0 ^bc^	6790.9 ± 1978.4 ^c^	6845.5 ± 1049.5 ^c^	8473.5 ± 1285.9 ^bc^	10,285.3 ± 1252.1 ^ab^	11,845.4 ± 246.9 ^a^	8766.1 ± 441.2 ^bc^	11,146.9 ± 398.7 ^ab^
C20:1	739.21 ± 57.09 ^a^	185.12 ± 30.15 ^d^	375.05 ± 75.76 ^c^	334.22 ± 20.77 ^cd^	573.12 ± 40.35 ^ab^	437.58 ± 93.47 ^bc^	558.99 ± 125.41 ^b^	243.83 ± 48.87 ^c^	421.38 ± 27.62 ^bc^	345.65 ± 33.88 ^bc^	1308.15 ± 174.78 ^a^	411.93 ± 31.9 ^bc^
C22:1n9	102.56 ± 10.84 ^b^	78.37 ± 19.08 ^bc^	101.88 ± 21.92 ^b^	53.73 ± 3.68 ^c^	206.03 ± 10.72 ^a^	89.46 ± 14.26 b^c^	101.37 ± 13.74 ^bc^	70.76 ± 11.06 ^c^	129.79 ± 9.99 ^ab^	53.75 ± 7.77 ^c^	142.75 ± 12.20 ^a^	65.45 ± 7.22 ^c^
C24:1	142.40 ± 10.99 ^a^	30.14 ± 8.57 ^b^	46.97 ± 6.11 ^b^	23.94 ± 1.82 ^b^	140.49 ± 9.95 ^a^	174.47 ± 60.16 ^a^	88.70 ± 23.42 ^b^	37.62 ± 4.68 ^c^	55.02 ± 1.61 b^c^	21.74 ± 3.77 ^c^	208.97 ± 9.08 ^a^	57.56 ± 15.48 ^bc^
ΣMUFA	6411.5 ± 1026.3 ^c^	8315.2 ± 1025.6 ^c^	15,893.3 ± 480.2 ^a^	14,663.1 ± 1646.9 ^ab^	10,850.7 ± 444.1 ^bc^	9443.1 ± 2726.3 ^c^	9295.2 ± 1292.8 ^c^	10,696.7 ± 1611.9 ^bc^	13,775.6 ± 1578.4 ^ab^	16,286.2 ± 337.8 ^a^	13,901.4 ± 1016.7 ^ab^	14,533.2 ± 344.5 ^a^
C18:2n6c	1937.4 ± 237.4 ^d^	4704.1 ± 459.1 ^bc^	8485.3 ± 644.5 ^a^	5946.8 ± 518.6 ^b^	4187.8 ± 942.2 ^bcd^	3387.6 ± 1181.8 ^cd^	1688.9 ± 382.9 ^c^	4751.0 ± 1420.1 ^ab^	6730.5 ± 514.3 ^a^	6200.2 ± 651.6 ^a^	2371.8 ± 478.3 ^bc^	7093.3 ± 1580.2 ^a^
C18:3n6	9.15 ± 0.85 ^c^	23.25 ± 9.79 ^bc^	24.85 ± 2.50 ^b^	45.55 ± 3.93 ^a^	28.61 ± 2.99 ^b^	18.44 ± 3.93 ^bc^	7.44 ± 0.39 ^c^	29.20 ± 4.06 ^ab^	30.15 ± 2.67 ^ab^	35.52 ± 3.61 ^a^	22.09 ± 3.66 ^b^	25.38 ± 4.04 ^ab^
C18:3n3	156.17 ± 13.53 ^c^	417.40 ± 54.99 ^bc^	1053.31 ± 196.72 ^a^	750.96 ± 160.4 ^ab^	762.58 ± 244.28 ^ab^	258.5 ± 81.21 ^c^	165.12 ± 35.98 ^b^	364.02 ± 92.67 ^b^	1518.20 ± 139.99 ^a^	1379.48 ± 426.25 ^a^	317.03 ± 44.06 ^b^	586.90 ± 10.96 ^b^
C20:2	176.90 ± 17.30 ^d^	320.20 ± 64.21 ^cd^	496.44 ± 30.24 ^b^	874.20 ± 61.52 ^a^	335.27 ± 48.27 ^c^	243.13 ± 57.05 ^cd^	351.56 ± 47.21 ^c^	421.57 ± 36.76 ^bc^	545.56 ± 97.66 ^bc^	927.92 ± 68.46 ^a^	323.09 ± 54.26 ^c^	621.13 ± 127.83 ^b^
C20:3n6	30.91 ± 4.48 ^c^	62.42 ± 17.41 ^b^	73.80 ± 2.67 ^b^	116.81 ± 17.24 ^a^	54.92 ± 5.38 ^bc^	42.45 ± 5.32 b^c^	43.53 ± 8.99 ^c^	63.67 ± 4.68 ^bc^	78.66 ± 10.38 ^b^	109.40 ± 14.18 ^a^	62.68 ± 4.84 b^c^	67.21 ± 7.32 ^bc^
C20:3n3	68.72 ± 10.22 ^c^	95.47 ± 14.67 ^abc^	139.79 ± 9.19 ^a^	138.46 ± 19.94 ^a^	121.78 ± 21.81 ^ab^	82.04 ± 16.76 ^bc^	120.19 ± 22.16 ^bc^	116.07 ± 9.05 ^bc^	219.75 ± 26.28 ^ab^	261.94 ± 76.63 ^a^	103.91 ± 9.92 ^c^	147.12 ± 4.26 ^bc^
C20:4n6	876.24 ± 72.01 ^a^	359.34 ± 75.63 ^b^	561.41 ± 36.90 ^b^	867.78 ± 64.5 ^a^	812.08 ± 56.56 ^a^	584.23 ± 111.85 ^b^	586.89 ± 118.81 ^b^	330.24 ± 75.40 ^b^	542.94 ± 46.38 ^b^	645.68 ± 99.1 ^b^	1753.32 ± 235.49 ^a^	507.46 ± 56.07 ^b^
C22:2	12.50 ± 1.81	22.97 ± 10.19	21.92 ± 2.02	23.76 ± 3.20	18.25 ± 1.24	14.65 ± 1.38	15.66 ± 1.55 ^b^	32.72 ± 4.37 ^a^	22.80 ± 2.37 ^b^	21.67 ± 5.39 ^b^	15.49 ± 0.81 ^b^	16.77 ± 2.03 ^b^
C20:5n3	494.37 ± 68.92 ^bc^	220.35 ± 39.34 ^c^	595.83 ± 91.71 ^b^	446.53 ± 55.94 ^bc^	992.87 ± 51.59 ^a^	781.91 ± 253.44 ^ab^	358.56 ± 62.34 ^b^	192.95 ± 54.65 ^b^	501.49 ± 25.85 ^b^	364.30 ± 21.92 ^b^	1520.38 ± 232.94 ^a^	553.87 ± 166.78 ^b^
C22:6n3	2817.9 ± 415.9 ^a^	203.5 ± 52.3 ^b^	872.5 ± 237.6 b	255.8 ± 25.9 ^b^	2930.9 ± 382.2 ^a^	2715.9 ± 902.1 ^a^	1741.25 ± 458.7 ^b^	157.5 ± 32.9 ^c^	665.29 ± 40.4 ^c^	193.6 ± 25.9 ^c^	5382.6 ± 483.5 ^a^	1024.6 ± 473.3 b^c^
ΣPUFA	6580.0 ± 519.7 ^b^	6429.1 ± 627.8 b	12,325.1 ± 497.9 ^a^	9466.6 ± 887.6 ^ab^	10,245.1 ± 1152.7 ^ab^	8128.8 ± 2541.6 ^b^	5079.1 ± 1126.1 ^b^	6459.1 ± 1712.6 ^b^	10,855.4 ± 776.6 ^a^	10,139.7 ± 1073.5 ^a^	11,872.5 ± 1531.5 ^a^	10,643.8 ± 1050.7 ^a^
Σn-3	3537.2 ± 504.6 ^ab^	936.8 ± 89.8 ^c^	2661.5 ± 211.4 ^bc^	1591.8 ± 248.1 ^c^	4808.2 ± 503.8 ^a^	3838.8 ± 1239.8 ^ab^	2385.1 ± 567.5 ^bc^	830.6 ± 186.3 ^c^	2904.7 ± 212.5 ^b^	2199.35 ± 474.9 ^bc^	7323.9 ± 769.2 ^a^	2312.5 ± 636.2 ^bc^
Σn-6	2853.7 ± 161.6 ^c^	5149.2 ± 479.1 ^bc^	9145.3 ± 608.2 ^a^	6976.9 ± 582.2 ^ab^	5083.4 ± 898.5 ^bc^	4032.7 ± 1280.3 ^c^	2326.8 ± 510.8 ^c^	5174.1 ± 1494.5 ^abc^	7382.3 ± 570.8 ^a^	6990.8 ± 644.1 ^ab^	4209.9 ± 719.2 ^bc^	7693.4 ± 1544.5 ^a^

Note: SFA: Saturated fatty acid; MUFA: Monounsaturated fatty acid; PUFA: Polyunsaturated fatty acid. The abbreviations stand for different regions. YY—Yongyan in Anhui province, PJ—Panjin in Liaoning province, HZ—Huzhou in Zhejiang province, JX—Jinxian in Jiangxi province, CZ—Changzhou in Jiangsu province, EZ—Ezhou in Hubei province. Values in the same row with different superscripts are significantly different (*p* < 0.05).

**Table 9:** The fatty acid composition in gonads of adult *Eriocheir sinensis* from different regions (mg/100 g wet weight).

	Male						Female					
	CZ	PJ	HZ	EZ	YY	JX	CZ	PJ	HZ	EZ	YY	JX
C14:0	-	-	-	-	-	-	117.99 ± 7.78 ^c^	111.03 ± 12.38 ^c^	169.75 ± 11.49 ^b^	94.34 ± 1.94 ^c^	215.56 ± 10.33 ^a^	105.7 ± 6.94 ^c^
C15:0	-	-	-	-	-	-	38.48 ± 0.34 ^c^	81.21 ± 10.11 ^ab^	89.78 ± 4.22 ^a^	60.46 ± 2.25 ^b^	64.88 ± 2.68 ^b^	42.17 ± 3.26 ^c^
C16:0	45.04 ± 4.31 ^c^	67.31 ± 10.20 ^c^	225.49 ± 20.53 ^a^	160.95 ± 13.22 ^b^	48.17 ± 8.68 ^c^	44.21 ± 4.21 ^c^	1698.15 ± 92.34 ^c^	2071.88 ± 77.61 ^b^	2716.76 ± 69.97 ^a^	1627.39 ± 54.99 ^c^	1909.47 ± 71.62 ^bc^	1750.53 ± 40.30 ^bc^
C17:0	-	-	-	-	-	-	57.31 ± 2.05 ^c^	94.99 ± 1.49 ^b^	123.87 ± 10.30 ^a^	80.87 ± 2.05 ^bc^	74.36 ± 3.50 ^bc^	53.73 ± 4.45 ^c^
C18:0	39.46 ± 3.92 ^c^	58.12 ± 8.97 ^bc^	123.78 ± 8.05 ^a^	103.88 ± 20.00 ^ab^	49.29 ± 6.62 ^c^	37.36 ± 2.01 ^c^	547.03 ± 28.86 ^c^	813.64 ± 14.41 ^b^	1167.53 ± 27.40 ^a^	627.74 ± 12.7 ^c^	540.28 ± 29.40 ^c^	598.15 ± 26.97 ^c^
C20:0	29.89 ± 2.06 ^a^	18.36 ± 2.15 ^b^	20.28 ± 1.25 ^b^	23.68 ± 0.57 ^ab^	20.80 ± 1.66 ^b^	19.31 ± 0.99 ^b^	38.64 ± 1.34 ^d^	89.49 ± 5.74 ^b^	118.41 ± 5.26 ^a^	70.25 ± 1.57 ^c^	46.33 ± 3.17 ^d^	39.06 ± 1.50 ^d^
C22:0	14.85 ± 0.86	15.61 ± 1.29	24.54 ± 1.18	20.12 ± 4.46	16.35 ± 0.58	18.23 ± 1.26	47.23 ± 4.53 ^c^	65.75 ± 3.06 ^ab^	78.19 ± 3.06 ^a^	46.29 ± 1.33 ^c^	59.66 ± 2.55 b^c^	71.66 ± 4.29 ^ab^
ΣSFA	129.24 ± 2.14 ^c^	159.4 ± 22.29 ^c^	394.08 ± 15.8 ^a^	308.63 ± 12.54 ^b^	134.61 ± 15.5 ^c^	119.11 ± 7.3 ^c^	2544.84 ± 125.24 ^c^	3328 ± 107.56 ^b^	4464.27 ± 108.64 ^a^	2607.36 ± 66.74 ^c^	2910.55 ± 118.74 ^bc^	2661 ± 76.96 ^c^
C16:1	33.16 ± 3.82 ^cd^	40.08 ± 2.65 ^bc^	62.01 ± 4.76 ^a^	52.59 ± 7.15 ^ab^	10.28 ± 0.81 ^d^	21.02 ± 0.53 ^d^	822.41 ± 81.19 ^d^	1761.89 ± 104.34 ^a^	1501.49 ± 41.72 ^ab^	1173.44 ± 50.18 ^bc^	1076.58 ± 94.23 ^cd^	1070.36 ± 8.83 ^cd^
C18:1n9c	79.89 ± 3.64 ^b^	66.47 ± 4.87 ^b^	276.15 ± 27.29 ^a^	233.07 ± 5.9 ^a^	84.12 ± 9.50 ^b^	81.76 ± 5.72 ^b^	2282.81 ± 136.66 ^c^	3647.52 ± 279.58 ^ab^	4393.19 ± 214.08 ^a^	2742.1 ± 136.18 ^c^	2619.72 ± 92.68 ^c^	2817.16 ± 155.04 ^bc^
C20:1	13.26 ± 1.58	11.65 ± 0.57	15.55 ± 2.41	13.68 ± 2.1	10.79 ± 0.31	9.52 ± 0.74	111.86 ± 7.59 ^b^	65.83 ± 6.01 ^cd^	125.5 ± 3.60 ^b^	54.55 ± 3.77 ^d^	215.08 ± 6.29 ^a^	82.53 ± 4.33 ^c^
C22:1n9	15.64 ± 1.69 ^c^	21.79 ± 1.93 ^bc^	30.25 ± 3.39 ^a^	25.47 ± 1.1 ^ab^	14.20 ± 2.04 ^c^	12.57 ± 1.39 ^c^	34.44 ± 4.14 ^c^	69.39 ± 3.75 ^b^	113.88 ± 2.82 ^a^	62.86 ± 3.71 ^b^	35.59 ± 1.22 ^c^	23.08 ± 1.86 ^c^
C24:1	73.39 ± 11.45	41.69 ± 14.3	85.87 ± 12.93	91.35 ± 19.73	83.47 ± 33.69	45.95 ± 10.72	14.40 ± 1.47 ^a^	10.35 ± 0.22 b^c^	16.09 ± 0.68 ^a^	10.26 ± 0.41 ^bc^	13.82 ± 0.76 ^ab^	7.72 ± 0.25 ^c^
ΣMUFA	215.33 ± 13.58 ^b^	181.67 ± 15.72 ^b^	489.82 ± 17.95 ^a^	422.82 ± 12.05 ^a^	201.88 ± 22.09 ^b^	170.82 ± 7.72 ^b^	3265.92 ± 208.6 ^b^	5554.99 ± 167.51 ^a^	6150.15 ± 248.41 ^a^	4043.22 ± 111.24 ^b^	3960.80 ± 173.44 ^b^	4000.84 ± 155.7 ^b^
C18:2n6c	26.24 ± 1.68 ^cd^	37.44 ± 1.83 ^c^	142.86 ± 5.39 ^a^	109.04 ± 5.17 ^b^	30.68 ± 2.40 ^cd^	20.74 ± 0.91 ^d^	1430.18 ± 88.68 ^c^	1871.2 ± 63.5 ^b^	2662.16 ± 115.42 ^a^	1592.32 ± 88.97 ^bc^	1845.95 ± 130.47 ^b^	1200.64 ± 50.82 ^c^
C18:3n3	-	-	-	-	-	-	133.62 ± 8.82 ^c^	516.1 ± 32.79 ^ab^	666.35 ± 12.51 ^a^	494.01 ± 71.07 ^b^	107.56 ± 10.40 ^c^	184 ± 16.61 ^c^
C20:2	13.11 ± 2.18 ^c^	14.55 ± 1.93 ^c^	30.49 ± 5.99 ^b^	48.56 ± 2.2 ^a^	15.49 ± 0.52 ^c^	18.89 ± 0.44 ^bc^	77.47 ± 4.60 ^c^	151.44 ± 24.27 ^b^	220.53 ± 14.55 ^a^	179.58 ± 3.78 ^ab^	74.76 ± 8.77 ^c^	119.63 ± 14.27 ^bc^
C20:3n6	-	-	-	-	-	-	10.43 ± 0.15 ^b^	35.99 ± 3.79 ^a^	43.75 ± 1.91 ^a^	38.23 ± 1.54 ^a^	13.07 ± 0.43 ^b^	15.88 ± 1.93 ^b^
C20:3n3	-	-	-	-	-	-	41.46 ± 3.53 ^c^	63.99 ± 3.56 ^bc^	135.47 ± 5.13 ^a^	79.61 ± 14.66 ^b^	32.44 ± 1.37 ^c^	45.38 ± 5.44 ^c^
C20:4n6	47.16 ± 2.43 ^bc^	35.65 ± 3.29 ^c^	125.07 ± 13.05 ^a^	105.6 ± 1.91 ^a^	69.27 ± 4.00 ^b^	42.05 ± 4.30 ^bc^	279.87 ± 8.62 ^b^	279.84 ± 22.24 ^b^	534.06 ± 40.69 ^a^	437.41 ± 51.53 ^a^	407.1 ± 27.59 ^ab^	287.24 ± 5.65 ^b^
C20:5n3	48.76 ± 0.20 ^bc^	41.86 ± 1.91 ^c^	68.46 ± 4.35 ^a^	45.18 ± 3.23 ^c^	58.37 ± 2.72 ^ab^	47.23 ± 1.97 ^bc^	618.36 ± 58.10 ^b^	325.28 ± 16.14 ^c^	739.33 ± 22.26 ^b^	412.30 ± 12.53 ^c^	1122.55 ± 58.68 ^a^	667.45 ± 28.59 ^b^
C22:6n3	55.72 ± 0.52 ^ab^	48.89 ± 0.44 ^ab^	49.92 ± 4.86 ^ab^	57.19 ± 2.13 ^a^	48.88 ± 0.54 ^ab^	42.86 ± 3.90 ^b^	635.18 ± 71.84 ^b^	180.85 ± 11.54 ^c^	859.67 ± 45.04 ^b^	222.98 ± 17.15 ^c^	683.35 ± 102.76 ^b^	1025.17 ± 52.48 ^a^
ΣPUFA	190.99 ± 2.72 ^cd^	178.41 ± 6.63 ^cd^	416.8 ± 18.83 ^a^	365.58 ± 4.22 ^b^	222.69 ± 6.95 ^c^	171.77 ± 10.82 ^d^	3226.57 ± 230.55 ^c^	3424.68 ± 80.4 ^bc^	5861.33 ± 232.88 ^a^	3456.43 ± 113.53 ^b^	4286.77 ± 327.95 ^b^	3545.39 ± 79.84 ^bc^
Σn-3	104.48 ± 0.59 ^ab^	90.75 ± 2.23 ^b^	118.38 ± 7.05 ^a^	102.38 ± 1.73 ^ab^	107.25 ± 2.56 ^ab^	90.09 ± 5.87 ^b^	1441.80 ± 155.53 ^c^	1086.21 ± 20.51 ^d^	2400.82 ± 73.67 ^a^	1208.90 ± 77.32 ^cd^	1945.90 ± 99.31 ^b^	1922 ± 75.66 ^b^
Σn-6	73.41 ± 1.47 ^cd^	73.10 ± 4.97 ^cd^	267.93 ± 15.46 ^a^	214.65 ± 6.54 ^b^	99.95 ± 4.87 ^c^	62.79 ± 5.19 ^d^	1737.47 ± 77.18 ^cd^	2187.04 ± 69.35 ^b^	3239.97 ± 152.76 ^a^	2067.96 ± 39.78 ^b^	2266.10 ± 124.1 ^bc^	1503.76 ± 49.86 ^d^

Note: SFA: Saturated fatty acid; MUFA: Monounsaturated fatty acid; PUFA: Polyunsaturated fatty acid. The abbreviations stand for different regions. YY—Yongyan in Anhui province, PJ—Panjin in Liaoning province, HZ—Huzhou in Zhejiang province, JX—Jinxian in Jiangxi province, CZ—Changzhou in Jiangsu province, EZ—Ezhou in Hubei province. Values in the same row with different superscripts are significantly different (*p* < 0.05).

## Data Availability

All data in this study are contained within the main manuscript and Supplementary Materials.

## Supplementary Materials

The following supporting information can be downloaded at https://www.mdpi.com/article/10.3390/ani15020243/s1: Table S1: Amino acid content per gram of protein; Table S2: The essential amino acid scores (EAAS) of different tissues of adult *Eriocher sinensis* from six different regions; Table S3: The chemical score (CS) of different tissues of adult *Eriocher sinensis* from six different regions.
