# Supplementary material for: Variability in Morphological Traits and Nutritional Profiles of Adult Eriocheir sinensis in Different Aquacultural Regions
Source: Animals (Basel). 2025 Jan 16;15(2):243. doi: 10.3390/ani15020243 (PMC11759138; doi:10.3390/ani15020243)
Supplement: Supplementary file 1 [file animals-15-00243-s001.zip › animals-3419011-supplementary.pdf]

Table S1. Amino acid content per gram of protein

	Amino acids	Content (mg/g prot)						WHO/FAO/UNU 2011	FAO egg protein model profile 1984
		CZ	PJ	HZ	EZ	YY	JX		
male musle	Thr	44.95±0.15	42.92±0.18	48.01±0.16	46.11±0.08	45.53±0.11	43.66±0.39	23	40
	Val	47.32±0.17	45.67±0.07	46.66±0.15	45.85±0.08	46.84±0.26	47.2±0.68	45	55
	Lys	80.40±0.11	80.86±0.14	79.83±0.27	79.96±0.45	80.45±0.49	79.26±0.30	59	70
	Ile	43.06±0.19	42.55±0.27	44.62±0.36	39.20±0.18	42.67±0.36	42.45±0.42	30	40
	Leu	76.13±0.07	77.26±0.07	79.23±0.36	74.80±0.11	75.97±0.07	74.13±0.78	22	35
	Met + Cys	26.75±0.23	16.88±0.27	22.18±0.23	18.18±0.22	21.84±1.61	35.54±3.36	38	60
	Phe + Tyr	80.83±1.05	75.16±0.21	83.27±0.89	79.08±0.57	78.86±0.64	83.51±2.61	39	50
	Total	399.43±0.85	381.3±0.52	403.82±1.18	383.19±0.55	392.14±1.88	405.75±3.40	256	350
female musle	Thr	46.62±0.05	44.35±0.06	45.43±0.21	46.48±0.20	46.32±0.91	45.47±0.10		
	Val	47.79±0.22	45.80±0.04	46.89±0.18	46.67±0.06	46.60±0.09	47.04±0.47		
	Lys	81.29±0.15	81.30±0.08	81.15±0.36	82.47±0.49	80.07±0.73	81.77±0.43		
	Ile	43.78±0.15	43.19±0.38	44.06±0.07	38.56±0.22	42.2±0.12	43.76±0.53		
	Leu	77.21±0.01	77.36±0.09	77.62±0.73	74.54±0.09	75.42±0.80	76.69±0.84		
	Met + Cys	22.11±0.18	26.83±0.15	22.73±0.22	15.98±0.72	28.27±4.46	25.76±1.63		
	Phe + Tyr	81.82±0.26	78.86±0.34	81.89±1.15	78.87±1.02	82.31±1.94	81.47±1.16		
	Total	400.62±0.23	397.68±0.16	399.77±2.52	383.57±1.57	401.2±4.11	401.95±0.68		
Male hepatopancreas	Thr	57.60±0.13	58.81±0.45	71.78±0.26	71.86±0.31	55.73±0.41	54.54±0.42		
	Val	61.31±0.50	58.71±0.31	56.60±0.09	59.26±0.19	60.40±0.66	62.22±0.42		
	Lys	80.53±1.88	67.39±0.63	59.55±0.26	58.68±0.09	71.80±0.90	77.36±0.42		
	Ile	47.85±0.27	45.43±0.56	36.10±0.08	37.24±0.29	45.92±0.97	48.20±0.46		
	Leu	84.76±0.11	80.86±0.51	66.08±0.08	68.39±0.18	83.72±0.27	86.92±0.83		
	Met + Cys	34.56±1.70	44.74±2.37	29.70±0.87	29.71±0.50	42.04±2.24	39.29±1.21		

	Phe + Tyr	97.07±2.98	85.68±1.68	60.73±0.47	64.88±0.79	94.78±0.36	97.24±1.99
	Total	463.68±2.29	441.61±4.41	380.54±0.65	390.01±1.08	454.39±1.02	465.77±0.92
female hepatopancreas	Thr	58.15±0.27	57.97±0.37	71.14±0.19	71.26±0.19	58.78±0.31	55.82±0.54
	Val	60.23±0.52	59.49±0.21	58.97±0.05	59.73±0.22	60.81±1.00	62.73±0.39
	Lys	79.13±0.87	73.80±0.36	60.04±0.07	58.37±0.14	78.06±0.34	77.47±1.42
	Ile	48.20±0.16	47.95±0.22	38.63±0.16	37.24±0.47	45.71±0.59	47.87±0.95
	Leu	83.87±0.26	83.27±0.48	66.63±0.11	67.8±0.35	84.13±0.46	87.31±1.63
	Met + Cys	32.72±0.84	31.40±2.88	23.94±0.96	35.43±0.65	38.86±0.26	41.23±2.69
	Phe + Tyr	88.46±0.34	88.81±0.18	65.93±1.45	62.68±1.64	83.09±0.81	86.06±3.79
	Total	450.75±2.08	442.69±1.96	385.28±1.85	392.51±2.4	449.45±1.87	458.5±7.78
testis	Thr	93.87±1.14	90.02±0.84	84.87±0.19	98.26±6.26	89.61±0.53	91.35±0.09
	Val	35.44±0.31	35.49±0.62	41.36±0.89	34.32±0.23	40.4±2.63	37.2±0.05
	Lys	46.36±0.87	49.42±1.03	60.64±0.36	40.61±2.16	51.83±3.31	49.2±0.13
	Ile	48.10±0.15	46.56±0.44	45.11±0.15	41.17±4.13	46.18±0.57	46.72±0.18
	Leu	69.60±0.40	66.09±0.21	66.93±0.05	57.63±5.33	68.27±0.9	70.36±0.16
	Met + Cys	21.49±1.91	18.38±0.41	170±0.78	14.60±0.83	18.93±2.19	18.57±0.12
	Phe + Tyr	66.01±1.77	60.99±0.16	64.14±0.33	59.17±17.36	62.51±2.37	66.29±0.50
	Total	380.87±1.78	366.94±1.86	380.06±1.66	345.76±23.28	377.73±0.85	379.7±0.39
ovary	Thr	59.19±0.04	59.05±0.22	58.45±0.05	60.41±0.15	60.39±0.08	59.99±0.02
	Val	66.37±0.04	64.70±0.12	64.71±0.11	63.83±0.20	65.43±0.07	65.70±0.09
	Lys	71.00±0.07	72.14±0.15	71.96±0.11	71.47±0.30	70.15±0.06	70.60±0.08
	Ile	48.74±0.06	47.9±0.06	48.26±0.23	45.70±0.18	46.66±0.03	47.69±0.16
	Leu	84.51±0.11	84.17±0.11	84.57±0.14	82.98±0.23	82.40±0.16	84.16±0.12
	Met + Cys	36.37±0.16	28.77±0.13	35.98±0.04	28.42±0.24	34.89±0.17	41.56±0.29
	Phe + Tyr	95.79±0.35	94.48±0.43	95.56±0.73	101.24±3.6	92.06±0.21	93.83±0.28

Total	461.98±0.45	451.22±0.62	459.51±1.04	454.07±3.21	451.98±0.26	463.53±0.45

Table S2. The essential amino acid scores (EAAS) of different tissues of adult *Eriocher sinensis* from six different regions

		**Male**						**Female**					
		**CZ**	**PJ**	**HZ**	**EZ**	**YY**	**JX**	**CZ**	**PJ**	**HZ**	**EZ**	**YY**	**JX**
Muscle	Threonine	1.80±0.01	1.72±0.01	1.92±0.01	1.84±0.01	1.82±0.01	1.75±0.02	1.86±0.01	1.77±0.01	1.82±0.01	1.86±0.01	1.85±0.04	1.82±0.01
	Valine	1.18±0.01	1.14±0.01	1.17±0.01	1.15±0.01	1.17±0.01	1.18±0.02	1.19±0.01	1.15±0.01	1.17±0.01	1.17±0.01	1.16±0.01	1.18±0.01
	Lysine	1.67±0.01	1.68±0.01	1.66±0.01	1.67±0.01	1.68±0.01	1.65±0.01	1.69±0.01	1.69±0.01	1.69±0.01	1.72±0.01	1.67±0.02	1.70±0.01
	Isoleucine	1.44±0.01	1.42±0.01	1.49±0.01	1.31±0.01	1.42±0.01	1.41±0.01	1.46±0.01	1.44±0.01	1.47±0.01	1.29±0.01	1.41±0.01	1.46±0.02
	Leucine	1.25±0.01	1.27±0.01	1.30±0.01	1.23±0.01	1.25±0.01	1.22±0.01	1.27±0.01	1.27±0.01	1.27±0.01	1.22±0.01	1.24±0.01	1.26±0.01
	SAA	1.16±0.01	0.73±0.01	0.96±0.01	0.79±0.01	0.95±0.07	1.55±0.15	0.96±0.01	1.17±0.01	0.99±0.01	0.69±0.03	1.23±0.19	1.12±0.07
	AAA	1.97±0.03	1.83±0.01	2.03±0.02	1.93±0.01	1.92±0.02	2.04±0.06	2.00±0.01	1.92±0.01	2.00±0.03	1.92±0.02	2.01±0.05	1.99±0.03
	Average	1.50±0.01	1.40±0.01	1.50±0.01	1.42±0.01	1.46±0.01	1.54±0.02	1.49±0.01	1.49±0.01	1.49±0.01	1.41±0.01	1.51±0.03	1.50±0.01
hepatopancreas	Threonine	2.3±0.01	2.35±0.02	2.87±0.01	2.87±0.01	2.23±0.02	2.18±0.02	2.33±0.01	2.32±0.01	2.85±0.01	2.85±0.01	2.35±0.01	2.23±0.02
	Valine	1.53±0.01	1.47±0.01	1.42±0	1.48±0	1.51±0.02	1.56±0.01	1.51±0.01	1.49±0.01	1.47±0	1.49±0.01	1.52±0.03	1.57±0.01
	Lysine	1.68±0.04	1.4±0.01	1.24±0.01	1.22±0	1.5±0.02	1.61±0.01	1.65±0.02	1.54±0.01	1.25±0	1.22±0	1.63±0.01	1.61±0.03
	Isoleucine	1.6±0.01	1.51±0.02	1.2±0	1.24±0.01	1.53±0.03	1.61±0.02	1.61±0.01	1.6±0.01	1.29±0.01	1.24±0.02	1.52±0.02	1.6±0.03
	Leucine	1.39±0	1.33±0.01	1.08±0	1.12±0	1.37±0	1.42±0.01	1.37±0	1.37±0.01	1.09±0	1.11±0.01	1.38±0.01	1.43±0.03
	SAA	1.5±0.07	1.95±0.1	1.29±0.04	1.29±0.02	1.83±0.1	1.71±0.05	1.42±0.04	1.37±0.13	1.04±0.04	1.54±0.03	1.69±0.01	1.79±0.12
	AAA	2.37±0.07	2.09±0.04	1.48±0.01	1.58±0.02	2.31±0.01	2.37±0.05	2.16±0.01	2.17±0	1.61±0.04	1.53±0.04	2.03±0.02	2.1±0.09
	Average	1.85±0.01	1.77±0.02	1.52±0	1.56±0	1.82±0	1.86±0	1.8±0.01	1.77±0.01	1.54±0.01	1.57±0.01	1.8±0.01	1.83±0.03
Gonad	Threonine	3.75±0.05	3.60±0.03	3.39±0.01	3.93±0.25	3.58±0.02	3.65±0	2.37±0	2.36±0.01	2.34±0	2.42±0.01	2.42±0	2.4±0
	Valine	0.89±0.01	0.89±0.02	1.03±0.02	0.86±0.01	1.01±0.07	0.93±0	1.66±0	1.62±0	1.62±0	1.6±0	1.64±0	1.64±0
	Lysine	0.97±0.02	1.03±0.02	1.26±0.01	0.85±0.04	1.08±0.07	1.03±0	1.48±0	1.5±0	1.5±0	1.49±0.01	1.46±0	1.47±0
	Isoleucine	1.6±0.01	1.55±0.01	1.5±0.01	1.37±0.14	1.54±0.02	1.56±0.01	1.62±0	1.6±0	1.61±0.01	1.52±0.01	1.56±0	1.59±0.01

Leucine	1.14±0.01	1.08±0	1.1±0	0.94±0.09	1.12±0.01	1.15±0	1.39±0	1.38±0	1.39±0	1.36±0	1.35±0	1.38±0
SAA	0.93±0.08	0.80±0.02	0.74±0.03	0.63±0.04	0.82±0.1	0.81±0.01	1.58±0.01	1.25±0.01	1.56±0	1.24±0.01	1.52±0.01	1.81±0.01
AAA	1.61±0.04	1.49±0	1.56±0.01	1.44±0.42	1.52±0.06	1.62±0.01	2.34±0.01	2.3±0.01	2.33±0.02	2.47±0.09	2.25±0.01	2.29±0.01
Average	1.52±0.01	1.47±0.01	1.52±0.01	1.38±0.09	1.51±0	1.52±0	1.85±0	1.8±0	1.84±0	1.82±0.01	1.81±0	1.85±0

Note: SAA-sulphur amino acids (methionine + cysteine); AAA-aromatic amino acids (phenylalanine + tyrosine). YY-Yongyan in Anhui province, PJ-Panjin in Liaoning province, HZ-Huzhou in Zhejiang province, JX-Jinxian in Jiangxi province, CZ-Changzhou in Jiangsu province, EZ-Ezhou in Hubei province.

Table S3. The chemical score (CS) of different tissues of adult *Eriocher sinensis* from six different regions

		**Male**						**Female**					
		**CZ**	**PJ**	**HZ**	**EZ**	**YY**	**JX**	**CZ**	**PJ**	**HZ**	**EZ**	**YY**	**JX**
Muscle	Threonine	1.12±0.01	1.07±0.01	1.20±0.01	1.15±0.01	1.14±0.01	1.09±0.01	1.17±0.01	1.11±0.01	1.14±0.01	1.16±0.01	1.16±0.02	1.14±0.01
	Valine	0.95±0.01	0.91±0.01	0.93±0.01	0.92±0.01	0.94±0.01	0.94±0.01	0.96±0.01	0.92±0.01	0.94±0.01	0.93±0.01	0.93±0.01	0.94±0.01
	Lysine	1.46±0.01	1.47±0.01	1.45±0.01	1.45±0.01	1.46±0.01	1.44±0.01	1.48±0.01	1.48±0.01	1.48±0.01	1.50±0.01	1.46±0.01	1.49±0.01
	Isoleucine	1.08±0.01	1.06±0.01	1.12±0.01	0.98±0.01	1.07±0.01	1.06±0.01	1.09±0.01	1.08±0.01	1.10±0.01	0.96±0.01	1.06±0.01	1.09±0.01
	Leucine	1.09±0.01	1.10±0.01	1.13±0.01	1.07±0.01	1.09±0.01	1.06±0.01	1.10±0.01	1.11±0.01	1.11±0.01	1.06±0.01	1.08±0.01	1.10±0.01
	SAA	0.79±0.01	0.50±0.01	0.65±0.01	0.53±0.01	0.64±0.05	1.05±0.10	0.65±0.01	0.79±0.01	0.67±0.01	0.47±0.02	0.83±0.13	0.76±0.05
	AAA	1.35±0.02	1.25±0.01	1.39±0.01	1.32±0.01	1.31±0.01	1.39±0.04	1.36±0.01	1.31±0.01	1.36±0.02	1.31±0.02	1.37±0.03	1.36±0.02
	Average	1.14±0.01	1.09±0.01	1.15±0.01	1.09±0.01	1.12±0.01	1.16±0.01	1.14±0.01	1.14±0.01	1.14±0.01	1.10±0.01	1.15±0.01	1.15±0.01
hepatopancreas	Threonine	1.44±0	1.47±0.01	1.79±0.01	1.8±0.01	1.39±0.01	1.36±0.01	1.45±0.01	1.45±0.01	1.78±0	1.78±0	1.47±0.01	1.40±0.01
	Valine	1.23±0.01	1.17±0.01	1.13±0	1.19±0	1.21±0.01	1.24±0.01	1.20±0.01	1.19±0	1.18±0	1.19±0	1.22±0.02	1.25±0.01
	Lysine	1.46±0.03	1.23±0.01	1.08±0	1.07±0	1.31±0.02	1.41±0.01	1.44±0.02	1.34±0.01	1.09±0	1.06±0	1.42±0.01	1.41±0.03
	Isoleucine	1.20±0.01	1.14±0.01	0.90±0	0.93±0.01	1.15±0.02	1.2±0.01	1.20±0	1.20±0.01	0.97±0	0.93±0.01	1.14±0.01	1.20±0.02
	Leucine	1.21±0	1.16±0.01	0.94±0	0.98±0	1.20±0	1.24±0.01	1.20±0	1.19±0.01	0.95±0	0.97±0	1.20±0.01	1.25±0.02
	SAA	1.02±0.05	1.32±0.07	0.87±0.03	0.87±0.01	1.24±0.07	1.16±0.04	0.96±0.02	0.92±0.08	0.70±0.03	1.04±0.02	1.14±0.01	1.21±0.08
	AAA	1.62±0.05	1.43±0.03	1.01±0.01	1.08±0.01	1.58±0.01	1.62±0.03	1.47±0.01	1.48±0	1.10±0.02	1.04±0.03	1.38±0.01	1.43±0.06
	Average	1.32±0.01	1.26±0.01	1.09±0	1.11±0	1.30±0	1.33±0	1.29±0.01	1.26±0.01	1.10±0.01	1.12±0.01	1.28±0.01	1.31±0.02
Gonad	Threonine	2.35±0.03	2.25±0.02	2.12±0	2.46±0.16	2.24±0.01	2.28±0	1.48±0	1.48±0.01	1.46±0	1.51±0	1.51±0	1.50±0
	Valine	0.71±0.01	0.71±0.01	0.83±0.02	0.69±0	0.81±0.05	0.74±0	1.33±0	1.29±0	1.29±0	1.28±0	1.31±0	1.31±0
	Lysine	0.84±0.02	0.9±0.02	1.10±0.01	0.74±0.04	0.94±0.06	0.89±0	1.29±0	1.31±0	1.31±0	1.30±0.01	1.28±0	1.28±0
	Isoleucine	1.20±0	1.16±0.01	1.13±0	1.03±0.1	1.15±0.01	1.17±0	1.22±0	1.20±0	1.21±0.01	1.14±0	1.17±0	1.19±0

Leucine	0.99±0.01	0.94±0	0.96±0	0.82±0.08	0.98±0.01	1.01±0	1.21±0	1.20±0	1.21±0	1.19±0	1.18±0	1.20±0
SAA	0.63±0.06	0.54±0.01	0.50±0.02	0.43±0.02	0.56±0.06	0.55±0	1.07±0	0.85±0	1.06±0	0.84±0.01	1.03±0.01	1.22±0.01
AAA	1.10±0.03	1.02±0	1.07±0.01	0.99±0.29	1.04±0.04	1.1±0.01	1.6±0.01	1.57±0.01	1.59±0.01	1.69±0.06	1.53±0	1.56±0
Average	1.09±0.01	1.05±0.01	1.09±0	0.99±0.07	1.08±0	1.08±0	1.32±0	1.29±0	1.31±0	1.3±0.01	1.29±0	1.32±0

Note: SAA-sulphur amino acids (methionine + cysteine); AAA-aromatic amino acids (phenylalanine + tyrosine). YY-Yongyan in Anhui province, PJ-Panjin in Liaoning province, HZ-Huzhou in Zhejiang province, JX-Jinxian in Jiangxi province, CZ-Changzhou in Jiangsu province, EZ-Ezhou in Hubei province.
